# A Modular Solid Phase Synthesis Approach for Glycocalix[4]Arene Derivatives and Their Multivalent Presentation on Ultrasmall Gold Nanoparticles.

**DOI:** 10.1002/chem.202500497

**Published:** 2025-06-27

**Authors:** Alisa Kayser, Kai Klein, Daria Babushkina, Anne Sakse, Gisele Mouafo Kenne, Ulla I.M. Gerling‐Driessen, Monir Tabatabai, Matthias Epple, Laura Hartmann

**Affiliations:** ^1^ Department of Organic Chemistry and Macromolecular Chemistry Heinrich Heine University Dusseldorf Universitätsstraße 1 Dusseldorf 40225 Germany; ^2^ Inorganic Chemistry and Center for Nanointegration Duisburg‐Essen (CENIDE) University of Duisburg Essen Universitätsstraße 7 Essen 45141 Germany; ^3^ Institute for Macromolecular Chemistry University of Freiburg Stefan‐Meier Straße 31 Freiburg i.Br. 79104 Germany

**Keywords:** bacterial inhibition, E. coli, glycocalix[n]arenes, gold nanoparticles, multivalency

## Abstract

Gold nanoparticles and calix[n]arenes are well‐established platforms for creating multivalent carbohydrate ligands that enhance binding avidity and selectivity toward carbohydrate‐recognizing receptors, such as bacterial lectins. In this study, we present a modular synthesis protocol for tailor‐made and (multi)functional glycocalix[4]arene derivatives using solid‐phase polymer synthesis. A calix[4]arene building block with a single carboxyl group on the lower rim and four nitro groups at the upper rim is introduced. This building block is attached to a solid support using standard solid phase peptide coupling conditions, followed by reduction of the upper rim nitro functionalities to yield four amine groups, that are further functionalized through solid‐phase polymer synthesis. Using this modular approach, we access a series of glyco‐calix[4]arene structures that are then further conjugated onto ultrasmall gold nanoparticles. Conjugation is promoted either via one or via four alkyne groups on the glycocalixarene structure, providing a handle to tune the overall valency of the final glyco[4]calixarene‐gold nanoparticle conjugates. Finally, the glycocalix[4]arene derivatives and conjugates are evaluated for their inhibitory potential against bacterial adhesion showing the importance of multivalent carbohydrate presentation to effectively block Escherichia coli (E. coli) adhesion.

## Graphical abstract

In this study, we present a calix[4]arene building block suitable for modular solid‐phase polymer synthesis and the preparation of functional glycocalix[4]arene. This allows us to incorporate carbohydrate ligands and further functionalities, such as alkynes at a specific position at the upper or lower rim of the calix[4]arene as demonstrated for the conjugation onto Au nanoparticles.

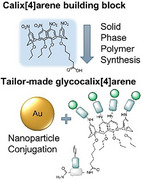

## 1 Introduction

Carbohydrates represent one of the major building blocks of life. Conjugated to proteins or lipids, they are essential components of the glycocalyx, a dense layer that surrounds every eucaryotic cell and plays a crucial role in mediating processes, such as cell communication, tumor metastasis or pathogen infection.^[^ ^1^ ^]^ While the underlying single carbohydrate‐protein interactions are usually weak, the multivalent binding of multiple epitopes of a multivalent carbohydrate ligand binding to one or more binding sites of a protein (e.g., lectins) is leading to an increased binding avidity thereby yielding strong and specific interactions.^[^ ^2^ ^]^ To study this so‐called glycoside cluster effect^[^ ^3^ ^]^ macromolecular scaffolds such as polymers,^[^ ^4^ ^]^ dendrimers,^[^ ^5^ ^]^ calixarenes^[^ ^6^ ^]^ and nanoparticles,^[^ ^7^ ^]^ have been employed to create synthetic multivalent carbohydrate ligands.

Gold nanoparticles are of great relevance in various biomedical applications^[^ ^8^ ^]^ due to their beneficial properties such as biocompatibility,^[^ ^8b^ ^]^ high cellular uptake properties^[^ ^8^, ^9^ ^]^ as well as high accumulation rates in tumor tissue.^[^ ^10^ ^]^ Furthermore, they exhibit unique magnetic, electronic, and optical properties, that can be useful for therapeutic and diagnostic applications.^[^ ^11^ ^]^ Glyco‐functionalized gold nanoparticles (g‐GNPs) are well‐established compounds to study and engage binding of carbohydrate recognizing proteins.^[^ ^7^, ^12^ ^]^ Besides the size^[^ ^7^, ^12^ ^]^ and shape of the gold core, also, the number and density of the carbohydrates on the particle surface^[^ ^7^, ^12^, ^13^ ^]^ as well as length and flexibility of the linker between the particle and carbohydrate^[^ ^7^, ^12^ ^]^ strongly influence the resulting protein binding and enhancement of binding through multivalent effects. Interestingly, it was shown that an excessive crowding of pendant carbohydrates on the nanoparticle surface can lead to sterically hindrance and thus no increase in binding through avidity is accomplished.^[^ ^12^, ^14^ ^]^ For these reasons, serval studies dedicated to the synthesis of well‐defined (ultrasmall) gold nanoparticles as well as synthetic pathways for a controlled surface functionalization have been reported.^[^ ^12^, ^15^ ^]^ Mainly, ultrasmall g‐GNPs with a metal core size of 2 nm or less are obtained via a direct synthesis approach (Brust synthesis) that gives access to ultrasmall thiol‐derivatized gold nanoparticles with high carbohydrate valency in one step.^[^ ^6^, ^15^, ^16^ ^]^ To enable a controlled variation of carbohydrate ligand density, additional noncarbohydrate functionalized components can be incorporated.^[^ ^12^, ^13^ ^]^ However, this approach meets limits due to different tendencies of chemisorption to the particle surface among thiol‐ligands. Furthermore, it must be considered that the additional modulating component can have interactions with the investigated target (e.g., carbohydrate recognizing protein), and that issues regarding water solubility have been reported using this method.^[^ ^15a^ ^]^ To overcome these disadvantages, van der Meer et al. reported a new strategy using multi‐thiol macromolecules allowing for the limitation of ligand crowding on the particle surface without the need of additional modulating components.^[^ ^17^ ^]^ This approach demonstrates the multiple conjugation to ultrasmall nanoparticle (< 2 nm) giving a handle to tune the degree of surface functionalization.

Herein, we present an advanced strategy to regulate the total number and density of carbohydrate ligands on the surface of ultrasmall gold nanoparticles (< 2 nm) by multivalent, covalent attachment of glycomacromolecules to the particles.^[^ ^18^ ^]^ In order to do so, we use calix[4]arenes as multivalent component for our macromolecular design. Similar to gold nanoparticles, calixarenes have been studied extensively as scaffolds for the multivalent presentation of carbohydrates and to study multivalent effects as well as their use in biomedicine, for example, in the inhibition of bacterial and viral adhesion as well as for the targeting of cancer cells.^[^ ^6^, ^19^ ^]^

**Figure 1:**
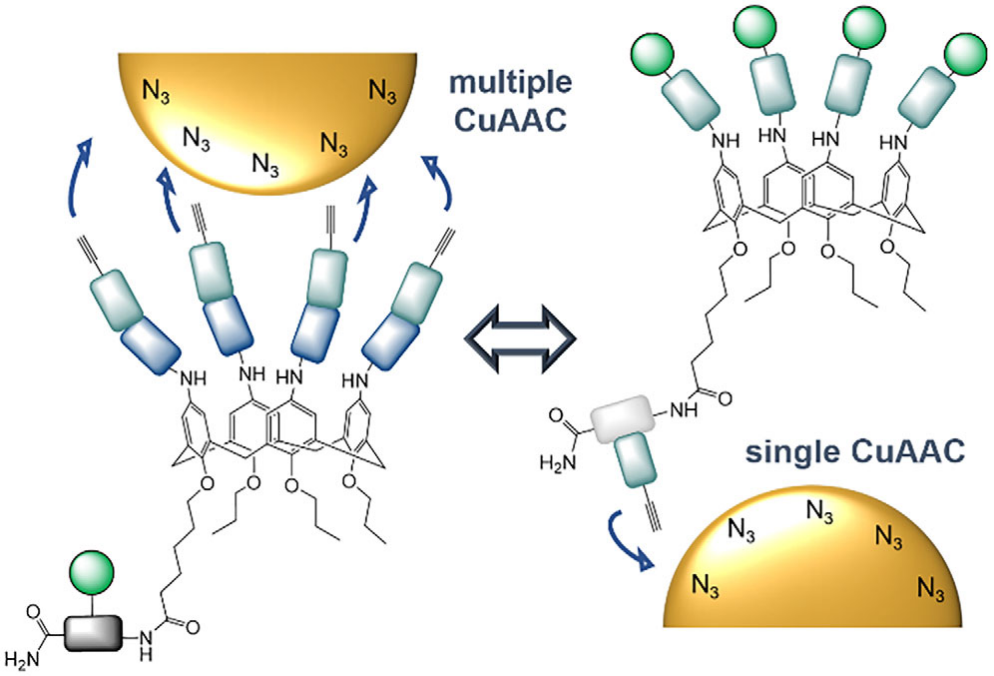
Schematic depiction of multiple versus single click approach depending on the ligand design.

We show a straightforward synthetic route using solid‐phase polymer synthesis to gain sequence‐defined glycocalix[4]arene (gCs) derivatives carrying four alkyne moieties at the upper rim and one carbohydrate residue at the lower rim or vice versa (Figure 1). This ligand design allows for a conjugation to azide functionalized gold nanoparticles via copper(I)‐catalyzed alkyne‐azide cycloaddition (CuAAC) through either just one or four alkyne groups simultaneously giving gC‐gold nanoparticle conjugates (gC‐GNPs) (Figure 1). This approach allows us to tune the density as well as the number of ligands on the particle surface. Based on the use of glycomacromolecules^[^ ^14^ ^]^ as inhibitors of bacterial adhesion, we investigate the synthesized gCs and gC‐GNPs for their inhibitory potential to bacterial adhesion.

## 2 Results and Discussion

### 2.1 Ligand Design and Synthesis of First Set of Glycocalix[4]Arenes C1‐C3

The focus of this work is the development and synthesis of tailor‐made and multifunctional gC derivatives. For this purpose, a solid‐phase synthesis previously established by Shuker et al,^[^ ^20^ ^]^ was combined with the solid‐phase polymer approach established in the Hartmann lab.^[^ ^21^ ^]^ First, a suitable calix[4]arene building block (CBB1) compatible with solid phase polymer synthesis was synthesized according to previously established procedures.^[^ ^20^ ^]^ CBB1 carries one carboxyl group at the lower rim enabling the selective attachment to the solid support via activation and amide coupling. The upper rim carries four nitro groups, which can be further modified once the CBB1 is attached to the solid support.

Attachment of CBB1 onto the solid support was performed on a TentaGel S RAM. The first three ethylene glycol diamine‐succinic acid 1‐(9H‐fluoren‐9‐yl)‐3,14‐dioxo‐2,7,10‐trioxa‐4,13‐diazaheptadecan‐17‐oic acid) (EDS) building blocks were coupled using previously established coupling conditions with DIPEA and PyBOP as activation reagents.^[^ ^21^, ^22^ ^]^ EDS introduces an ethylene glycol as the main chain motif leading to higher hydrophilicity and therefore facilitates the later analysis of the compound by HPLC‐MS. Next, CBB1 was coupled to the EDS_3_ oligomer using PyBOP and DIPEA as coupling reagents. After microcleavage, analysis of the formed product by HPLC‐MS and MALDI‐TOF‐MS confirms that CBB1 is coupled with high yields at these standard coupling conditions (see Supporting Information for details). After successful attachment of CBB1 to the solid support, the nitro groups of CBB1 were reduced to amines by treatment with an excess of Sn(II)Cl_2_ dihydrate followed by excessive washing with methanol, DMF, and DCM. We observed that the successful reduction of all four nitro groups strongly depends on the reaction conditions, especially the reaction temperature. In contrast to previously reported protocols, initial attempts at room temperature showed the formation of by‐products due to insufficient reduction of all nitro groups (data not shown).^[^ ^20^ ^]^ We found that best results were obtained when the reduction was performed with a high excess of Sn(II)Cl_2_ dihydrate in NMP at 30° C for 24 hours, which we confirmed by HPLC‐MS, HR‐ESI, and MALDI‐TOF‐MS (see Supporting Information for analytical data and further details on the reaction conditions). NMP was established as an optimal solvent since harsher reaction conditions, such as increased reaction temperature and longer reaction time yielded a by‐product that presumably occurred due to formylation in DMF (see Supporting Information).

**Scheme 1:**
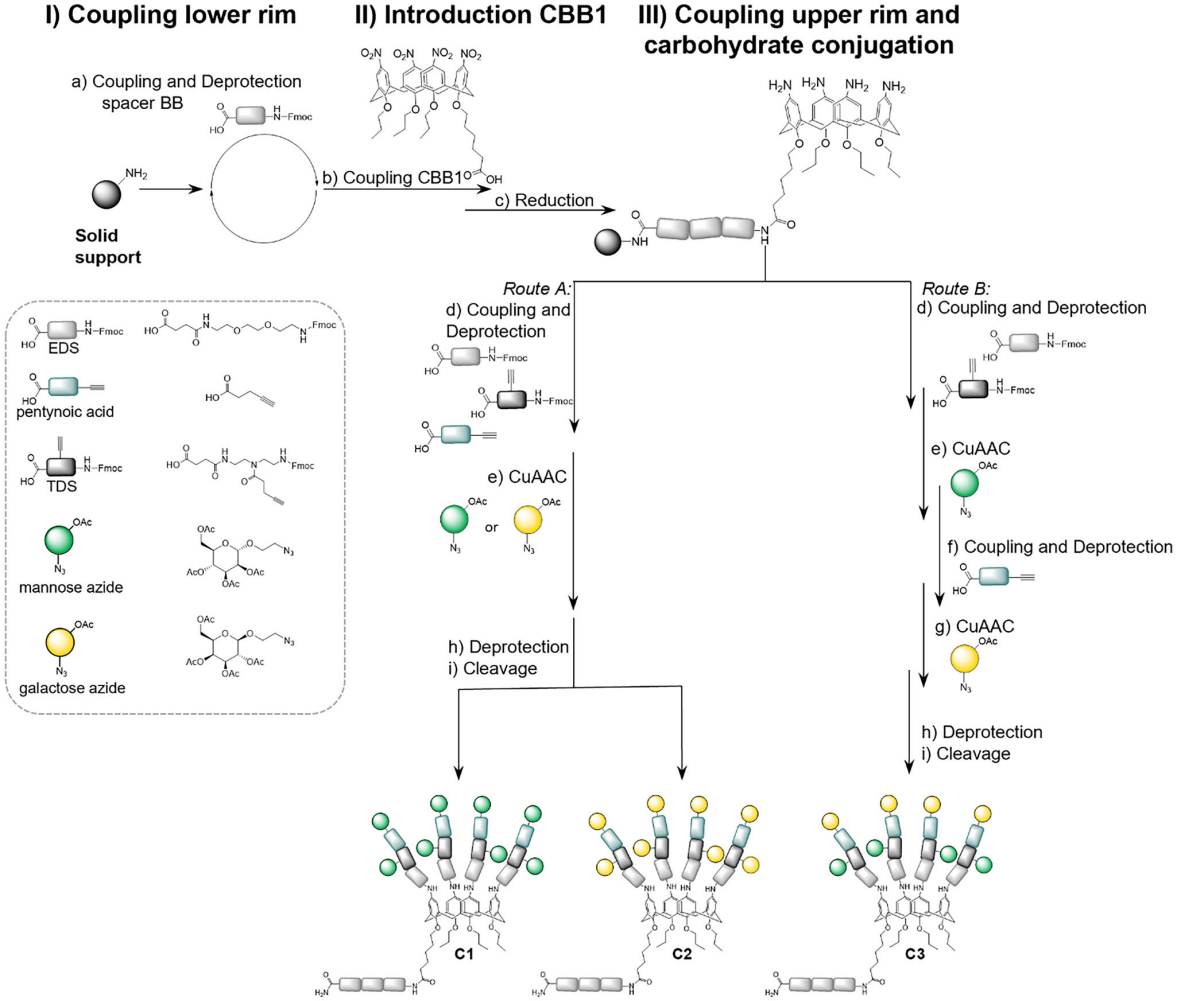
Solid‐phase synthesis of glycocalix[4]arenes C1‐C3. Reaction conditions: a) Coupling conditions: 5eq. building block, 5 eq. PyBOP, 10 eq. DIPEA, DMF, 1 hour; for Fmoc deprotection: 20 Vol.% piperidine in DMF was used. b) Coupling conditions: 3 eq. CBB1, 5 eq. PyBOP, 10 eq. DIPEA, DMF, overnight. c) 25 eq. Sn(II)Cl_2_ dihydrate per nitrogen group, NMP, 24 hours, 30° C. d) and f) Coupling conditions: 5eq. building block per amine, 5eq. PyBOP per amine, 10 eq. N‐methyl morpholine per amine, DMF, 1 hours; for Fmoc deprotection 20 Vol.% piperidine in DMF was used. e) and g) 2 eq. carbohydrate azide per alkyne, 20 mol % CuSO_4_, 20 mol % NaAsc, DMF/water, overnight. h) 0.2 M sodium methanolate, 30 minutes. i) 95/5/5 (vol.%) TFA/TIPS/DCM, 1 hour.

**Figure 2:**
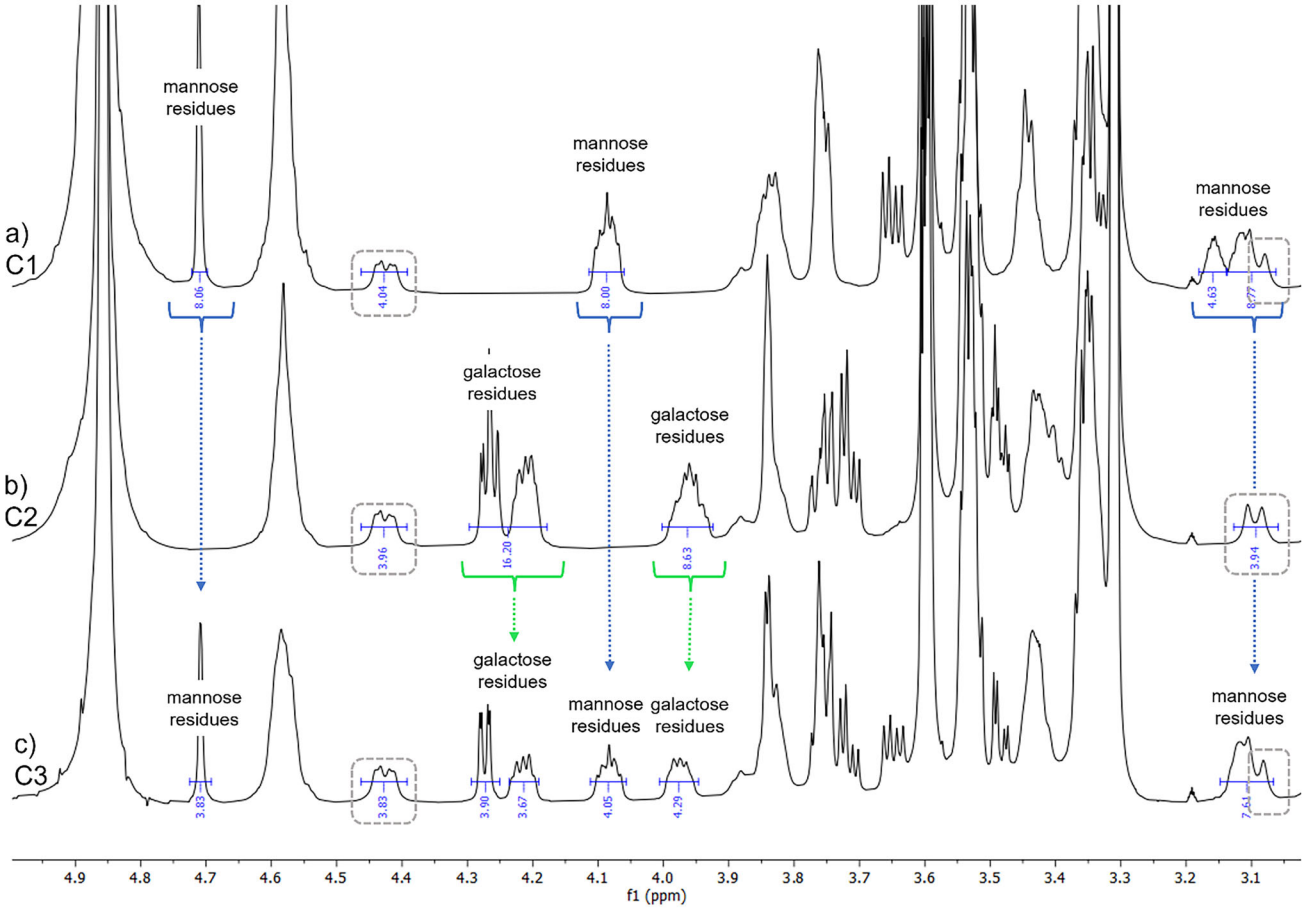
Excerpt from ^1^H‐NMR (600 Hz) spectra of compounds C1‐C3 in methanol‐_d4_/D_2_O. For full spectra see Supporting Information. Signals from calix[4]arene are marked in gray boxes.

After successful formation of the amine functionalities, further building blocks can be coupled via solid phase peptide or polymer synthesis to the upper rim of the calix[4]arene backbone. For different Fmoc‐protected building blocks as well as amino acids, N‐methyl morpholine, and PyBOP as coupling reagents showed the highest coupling efficiency (see Supporting Information for further details). For the targeted gC derivatives C1‐C3, the upper rim was functionalized with an EDS followed by a triple‐bond diethylenetriamine‐succinic acid, 1‐(fluorenyl)‐3,11‐dioxo‐7‐(pent‐4‐ynoyl)‐2‐oxa‐4,7,10‐triazatetra‐decan‐14‐oic acid (TDS building block that introduces an alkyne moiety).^[^ ^21^ ^]^ The incorporation of the alkyne‐functionality allows in the next step for the conjugation of azide functionalized carbohydrate residues, here mannose and galactose, via copper‐catalyzed azide‐alkyne cycloaddition (CuAAC). At this point, different synthetic pathways are possible to either obtain octavalent homo‐ or heteromultivalent derivatives, as shown in Scheme 1. In route A, pentynoic acid is coupled next to introduce a further, terminal alkyne functionality and subsequently all sugar moieties can be conjugated simultaneously to obtain homomultivalent derivatives C1‐C2. Alternatively, in route B, different azide‐carbohydrates can be conjugated in a sequential way to accomplish the heteromultivalent derivative C3.^[^ ^23^ ^]^ The glyco‐calix[4]arene structures were purified using preparative HPLC (see Supporting Information) and characterized by ^1^H‐NMR (Figure 2) and HPLC‐MS and MADLI‐TOF‐MS (see Supporting Information). The ^1^H‐NMR spectra of all three compounds show the signals of methylene bridge of calix[4]arene motif, which are marked in grey (Figure 2). Additional integrated signals can be assigned to the carbohydrate residues. Comparing the spectra chemical shift of the carbohydrate‐related signals can be recognized and validates the mannose conjugation in the case of C1 or galactose residues for C2. Additionally, the intensity of eight protons for each of the carbohydrate‐related peaks confirms the successful octavalent conjugation of the carbohydrates to the scaffold. For the heteromultivalent structure C3, signals of both galactose and mannose residues can be observed when compared to the homomultivalent structure, evidencing the successful synthesis of the heteromultivalent compound C3 with four mannose and galactose residues each.

### 2.2 Synthesis of Second Set of Glycocalix[4]Arenes C4‐C7

**Scheme 2:**
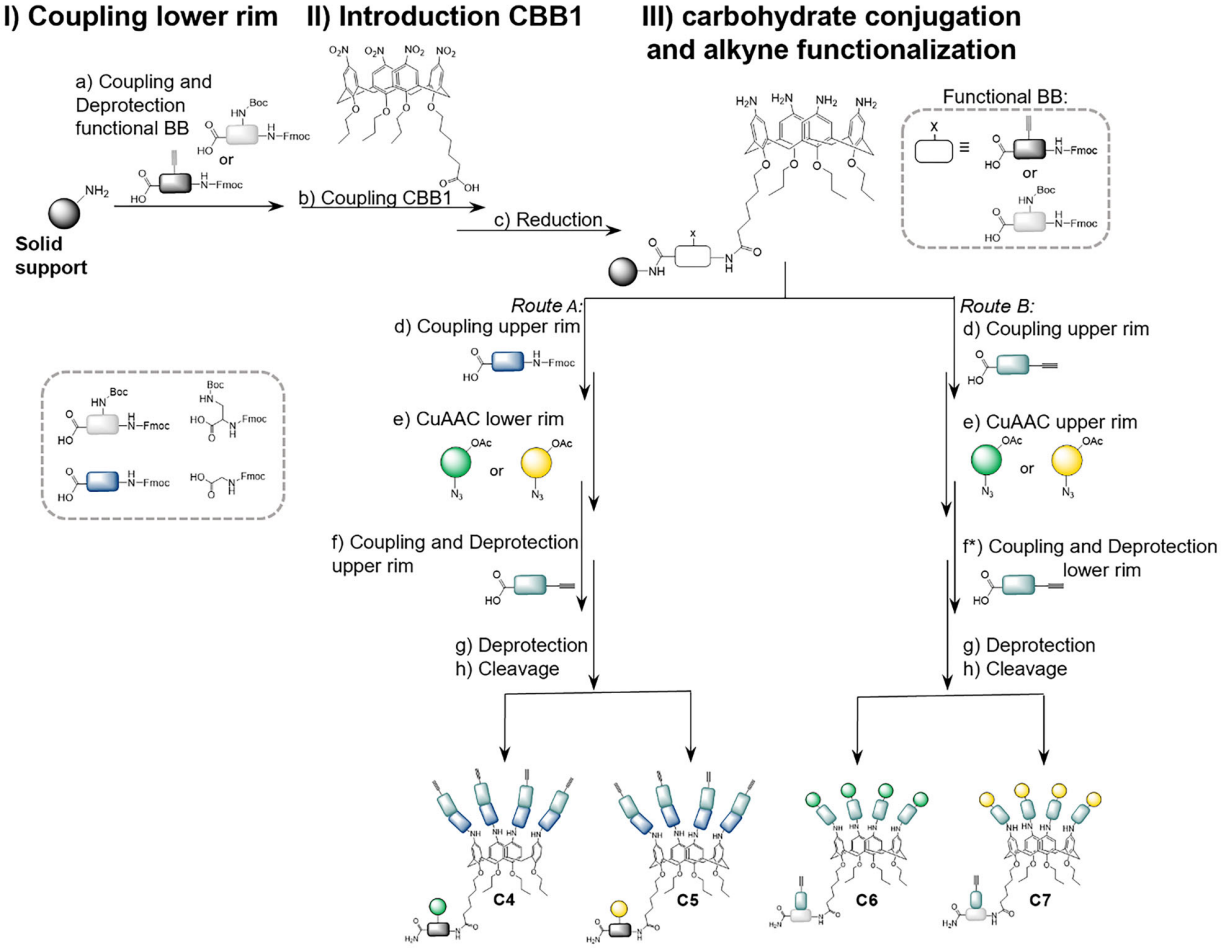
Solid‐phase synthesis of glycocalix[4]arenes C4‐C7. Rection conditions: a) 5eq. building block, 5 eq. PyBOP, 10 eq. DIPEA, DMF, 1 hour; for Fmoc deprotection 25% piperidine solution was used. b) 3 eq. CBB1, 5 eq. PyBOP, 10 eq. DIPEA, DMF, overnight. c) 25 eq. Sn(II)Cl_2_ dihydrate per nitrogen group, NMP, 24 hours, 30° C. d) and f) 5eq. Building block per amine, 5eq. PyBOP per amine, 10 eq. N‐methyl morpholine per amine, DMF, 1h¸ for Fmoc deprotection 25% piperidine solution was used. e) 2 eq. carbohydrate azide per alkyne, 20 mol % CuSO_4_, 20 mol % NaAsc, DMF/water, overnight. f*) 5eq. building block, 5 eq. PyBOP, 10 eq. DIPEA, DMF; for Boc deprotection 4 M HCl in Dioxan was used. g) 0.2 M sodium methanolate, 30 minutes. h) 95/5/5 TFA/TIPS/DCM (vol.%), 1 hour.

For the second set of gCs we aimed to incorporate additional alkyne moieties as second functionality into our ligand design. The exhibition of additional alkyne moieties will give access to further conjugation to larger scaffolds like azide‐bearing nanoparticles via CuAAC. We reasoned that the increased multivalency and size of the carbohydrate conjugates would result in improved receptor binding, thereby leading to the development of potent pathogen adhesion inhibitors. Several studies showed that the number and density of carbohydrates on the nanoparticle surface effect the binding avidity of the nanoparticles to lectins.^[^ ^7^, ^12^, ^13^, ^14^ ^]^ We therefore endeavored two different gC derivatives with either four alkyne groups at the upper rim and one carbohydrate group at the lower rim (C4,C5) or vice versa (C6,C7). This approach allows us to conjugate the calix[4]arene ligands to the nanoparticle via CuAAC in either multiple (C4,C5) or single (C6,C7) way and thereby give a handle to the adjust number and density of carbohydrates on the nanoparticle surface. For the synthesis of the alkyne‐gC derivative C4‐C7, previous reaction conditions were applied again, while the choice of building blocks and the order of their assembly was altered according to the final ligand structure design. Specifically, for the derivatives C4,C5 (Scheme 2, route A) instead of the spacing building blocks (EDS), now one alkyne carrying TDS building block was coupled as an initial building block, immediately followed by coupling of the CBB1 and subsequent reduction of the nitro groups. No notable evidence of a decreased coupling efficiency of CBB1 to TDS compared to EDS was observed. However, we noticed that one prerequisite is that no free amines at the upper rim of the calixarene are present while the sugar azide is attached to TDS at the lower rim via CuAAC as this affected the conjugation efficiency during the click reaction. This could be attributed to a complexation of the catalyzing copper ions by the free amines. Neither is it possible to conjugate the carbohydrate residue before the reduction step has been performed, as the glycosidic bond is not stable under the applied reduction conditions. For these reasons, we decided to react the interfering amines at the upper rim with Fmoc‐glycine prior to conjugation of carbohydrate azides. Finally, the remaining alkyne moieties were incorporated by terminal coupling of pentynoic acid to each of the glycine.

**Figure 3:**
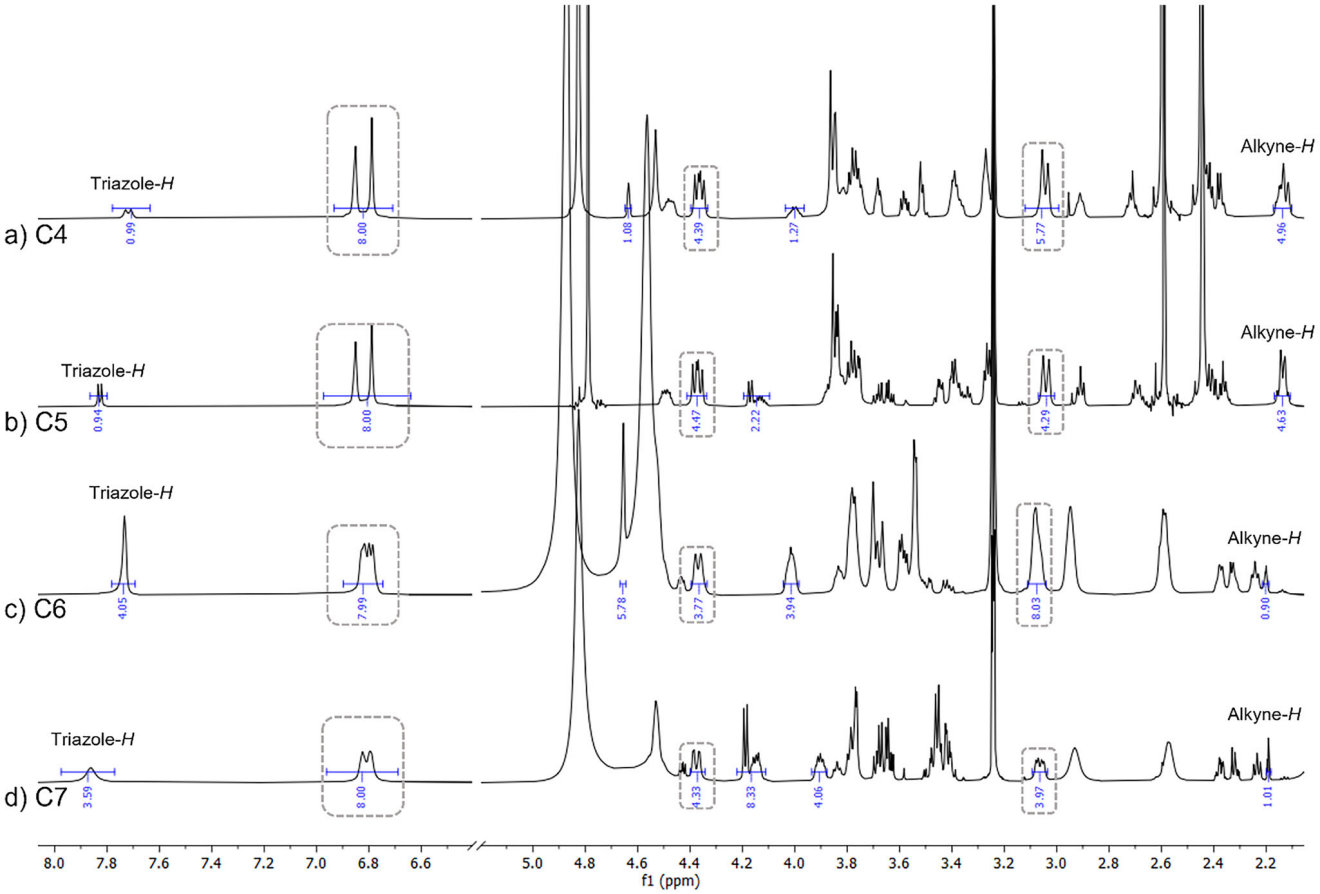
Excerpts from ^1^H‐NMR (600 MHz) spectra of compounds C4‐C7 in methanol‐*d4*/D_2_O. For full spectra see Supporting Information. Signals from calix[4]arene are marked in gray boxes.

The synthesis of C6,C7 was performed in a slightly altered fashion (Scheme 2, route B). Here an orthogonally Fmoc‐ and Boc‐protected diamino propionic acid was coupled instead of TDS as the first building block. Importantly, the Boc group on one amine of the diamino propionic acid can be selectively removed on solid support after functionalization of the upper rim, using 4 M HCl in dioxan. This is followed by coupling of pentynoic acid at the lower rim, which yields the required alkyne moiety at the lower rim without noteworthy effect on the residual structure. Using this method, the desired derivatives C6 and C7 were obtained efficiently. The crude products were purified using preparative HPLC (see Supporting Information) and all structures were analyzed by ^1^H‐NMR (Figure 3), HPLC‐MS, and MALDI‐TOF‐MS (see Supporting Information). Figure 3 shows the ^1^H‐NMR spectra of the alkyne functionalized gCs C4‐C7. Marked in gray are the signals assigned to the calix[4]arene motif for all derivatives. In addition, characteristic signals are assigned and integrated for the different derivatives. The downfield shifted signals are attributed to the triazole proton and can be found with an intensity of one proton for C4 and C5 and an intensity of four protons for C6 and C7, confirming the conjugation of one or four carbohydrate residues via CuAAC, respectively. Furthermore, all spectra show the expected signals for the mannose or galactose residues. Also, the signals for the alkyne protons are highlighted in the spectra, and integrals of either one for C4 and C5 or four for C6 and C7 further confirm successful synthesis of the targeted structures. In summary, we successfully established a versatile synthesis route for various calix[4]arene derivatives using solid‐phase protocols, as demonstrated by the synthesis of a series of gCs.

### 2.3 Attachment of C4‐C7 to Ultrasmall Gold Nanoparticles and Characterization of gC‐GNP

**Figure 4:**
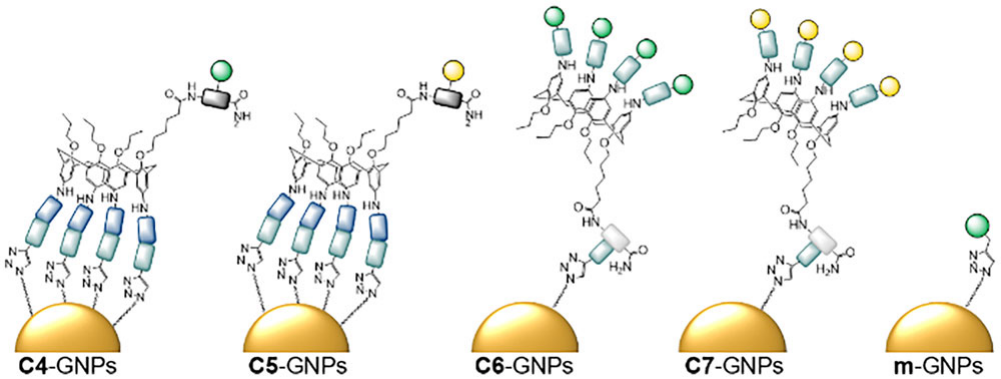
Schematic depiction and nomenclature of mannose‐calixarene gold‐nanoparticles C4‐GNPs, C5‐GNPs, C6‐GNPs, and C7‐GNPs and reference structure m‐GNPs.

The functionalization of ultrasmall azide‐bearing gold nanoparticles (a‐GNPs) allowing for an efficient way of covalent nanoparticle functionalization via CuAAC was demonstrated recently by Klein et al.^[^ ^24^ ^]^ Here, we wanted to test if the alkyne‐bearing gCs C4‐C7 are suitable for the conjugation to azide‐functionalized ultrasmall GNPs following this protocol. As a reference, we used propargyl mannose without calix[4]arene backbone for conjugation in an analogous fashion, giving mannose‐gold nanoparticles (m‐GNPs, Figure 4). The GNP conjugates (C4‐GNPs–C7‐GNPs and m‐GNPs, Figure 4) were characterized using differential sedimentation centrifugation (DSC, Table 1 and Supporting Information), ^1^H‐NMR and UV‐Vis spectroscopy (see Supporting Information).

**Table 1:** Hydrodynamic diameter d_H_ and degree of carbohydrate functionalization of the different gC‐GNPS as determined by DCS. The concentration of carbohydrates was determined by sulfuric acid phenol method (measurements were performed in triplicates, the error is the standard deviation of the mean value, see Supporting Information for further details) and the carbohydrate to nanoparticle ratio was calculated using the molar concentration of nanoparticles as derived by AAS (see Supporting Information for further details).

Ligand	d_H_ [nm]	C[carbohydrate] [µM]	N[carbohydrates molecules per nanoparticle]^[^ ^a^ ^]^
N3‐GNPs	1.5 ± 0.2	‐	‐
C4‐GNPs	1.4 ± 0.4	658.2 ± 65	16 ± 2
C5‐GNPs	1.5 ± 0.7	236.8 ± 44	6 ± 1
C6‐GNPs	1.3 ± 0.2	886.8 ± 30	129 ± 4
C7‐GNPs	1.5 ± 0.5	601.1 ± 65	88 ± 9
m‐GNPs	n.d.	472 ± 44	11 ± 1

^[a]^ N(carbohydrates molecules per nanoparticle) = c(carbohydrates)/c(NP); for c(NP) see Supporting Information.

They were found to be stable in water as well as in PBS buffer for several months. After approximately six months, we observed some precipitation in the samples, which was presumably attributed to nanoparticle aggregation. Nevertheless, the samples were successfully restored by the addition of 1 M NaOH and ultrasonification.

The absence of surface plasmon resonance in the UV‐Vis spectra, which can be observed for gold nanoparticles with a radius of more than 2 nm, indicated that no larger (plasmonic) gold nanoparticles were present (see Supporting Information).^[^ ^25^ ^]^ This finding was additionally supported by disc centrifugal sedimentation (DCS), which revealed that the measured hydrodynamic radii of the glycocalixarene‐gold nanoparticle conjugates were consistently smaller than 2 nm (Table 1). Furthermore, ^1^H‐NMR spectroscopy indicated the successful conjugation by general broadening of the ligand signals (see Supporting Information).^[^ ^26^ ^]^ However, to quantitatively assess the surface functionalization of gC‐GNPs, these methods were not suitable. The sulfuric acid phenol test was used as a colorimetric method in combination with atomic absorption spectroscopy (AAS) to determine the total carbohydrate concentration of the samples. AAS allows to determine the exact concentration of goldin the given dispersion, which can be further converted to the molar concentration of nanoparticles in the dispersion.^[^ ^24^ ^]^ Accordingly, the ratio of the molar concentration of carbohydrate molecules to the molar concentration of nanoparticles gives the average number of carbohydrates present on each particle. As shown in Table 1, the obtained results are in good agreement with the theoretically expected values and show that the average number of carbohydrates is around 20 times higher for C6‐GNPs in comparison to C4‐GNPs. For the galactose‐functionalized analogous, we observed a similar trend. Here the amount of carbohydrates was found to be around 15 times higher for C7‐GNPs compared to C5‐GNPs. Surprisingly, m‐GNPs showed a similar amount of carbohydrates in the sulfuric acid‐phenol test as C4‐GNPs. This might be due to shorter linker length causing reduced click efficiency. In summary, the attachment of gCs either through one or four alkyne groups allows to effectively tune the degree of functionalization on the GNPS and thus to control the overall carbohydrate functionalization for resulting gC‐GNPs. The number of carbohydrate molecules per nanoparticle is well in line with earlier results on cysteine‐conjugated peptides on ultrasmall gold nanoparticles.^[^ ^27^ ^]^

### 2.4 Bacterial Adhesion‐Inhibition Assay

Based on their carbohydrate functionalization and previous studies in literature using both gC as well as GNPs as inhibitors of viral and bacterial infections,^[^ ^28^ ^]^ we tested the inhibitory potential of the derived gCs C1‐C3 as well as gC‐GNPs C4‐C7 in an adhesion‐inhibition assay with the 1‐fimbriated *E. Coli* strain pPKL1162 expressing GFP. FimH receptors on the surface of the *E. coli* bacteria show specific binding to α‐D‐mannopyranoside and adhere well on mannan‐coated micro titer plates.^[^ ^29^ ^]^ This binding can be inhibited by mannose‐based compounds such as methyl mannose (MeMan) at high enough concentrations resulting in a decreased number of bacteria on the microtiter plate surface. Thus, the inhibitory potential can be observed by quantifying the GFP fluorescence of the E‐coli bacteria that can still adhere to the mannan‐coated micro titer plate surface. When applying the examined ligands in a serial dilution series of decreasing concentrations, the half maximum inhibition concertation (IC_50_) values can be determined. To achieve a better comparability among different assays, the IC_50_ values can be referenced with the IC_50_ value obtained for MeMan of each single experiment leading to the referenced inhibitory potential (RIP). This can further be corrected by the valency of binding mannose epitopes for the investigated multivalent structures (RIP_VC_).^[^ ^22^, ^30^ ^]^

Table 2 summarizes the determined IC_50_ and RIP values for C1 and C3 after incubation for 45 minutes at 37° C. For C1 a distinct increase of the inhibitor potential by a factor of 13 per binding sugar can be observed in comparison to MeMan. Additionally, glycomacromolecules with hydrophobic backbone motifs that were previously reported by our group exhibit lower RIP_VC_ values.^[^ ^30^ ^]^ These findings indicate that not only the incorporation of a hydrophobic motif is beneficial, but also that the cluster‐like presentation of the sugar epitopes on the calixarene backbone has a favorable effect on bacteria binding. Interestingly, for the heteromultivalent structure C3 an even higher increase by a factor of 19 per binding sugar was observed. This is consistent with the so‐called heterocluster effect, which was previously reported in several studies for various model lectins like Concanavalin A.^[^ ^23^, ^29^, ^31^ ^]^ It was proposed that the heterocluster effect can be accounted for by different processes promoted by the nonbinding sugar in heteromultivalent glycoconjugates, such as subside binding, structural preorganization, and sterical effects.^[^ ^32^ ^]^ As anticipated, for the control structure C2 with galactose residues, no IC_50_ could be determined.

**Table 2:** IC_50_, RIP and RIP_VC_ values for the first set of glyco‐calixarenes determined by bacterial adhesion‐inhibition assay on mannan coated plates. All measurements were performed in triplicates.

Ligand	IC_50_ [µM]	RIP	RIP_VC_
C1	97 ± 45	102 ± 55	13 ± 7
C2	n.d^[^ ^a^ ^]^	‐	‐
C3	128 ± 39	77 ± 30	19 ± 7

^[a]^ For the galactosyl functionalized control structure C2 no IC_50_ value could be determined.

Despite the increased inhibitory potential of C1 and C3, it is not likely to assume that one single gC is able to bind more than one FimH receptor at the same time simply due to their size and spatial arrangement. However, the multivalent display of several gCs on larger scaffolds, such as GNPs in the obtained gC‐GNP conjugates, could lead to a further enhanced inhibitory potential. By bridging multiple receptors at the same time, binding avidity might increase further. Since this effect was not expected for the sterically demanding gCs (C1‐C3), we conjugated only the sterically less demanding gCs (C4‐C7) to the GNPs. However, when performing the adhesion‐inhibition assay with gC‐GNPs we observed large scattering of the fluorescent measurement, which hampered the assessment of IC_50_ values (data not shown). For the mannose‐containing structures C4 and C6‐GNP conjugates we observed binding, even though no IC_50_ values could be determined. In contrast, no binding was detected for the monovalent mannose GNP conjugate m‐GNP in all experiments. For MeMan as monovalent positive control that was included in each single experiment, we do observe binding. Thus, potentially in the monovalent GNP control, the mannose ligands might be sterically too hindered to bind with the receptor, as similar observations have been made for other GNP systems.^[^ ^7c^ ^]^ We hypothesize that the fluctuations observed in our experiment can be accounted for by the formation of bacteria‐nanoparticle agglomerates. To further investigate the samples and to support this hypothesis, we engaged fluorescence microscopy (see Supporting Information). Indeed, we found large fluorescent agglomerates after incubating *E. coli* bacteria suspension in PBS buffer for 45 minutes with C6‐GNPs. Unexpectedly, the formation of bacteria agglomerates was also observed for bacteria suspension that was not incubated with any glyco conjugate, even though to a smaller extent. Interestingly, after incubation with C1 the formation of agglomerates was notably decreased. Future studies will explore this further, also by using other binding assays such as a fluorescence competition assay for carbohydrate‐functionalized GNPs.^[^ ^7c^ ^]^ Nevertheless, we believe that already our first proof‐of‐concept study for the combination of gCs and GNPs to derive inhibitors of bacterial adhesion shows the potential of leveraging this dual multivalent presentation.

## 3 Conclusion

In this study we successfully developed and established solid‐phase synthetic protocols for the modular preparation of several gCs, that also allows the incorporation of additional functional groups such as alkyne moieties at both rims of the gC structures. In a second step, gCs were conjugated to ultrasmall GNPs via CuAAC via either one or four pending alkyne residues providing a handle to adapt the number of carbohydrate ligands on the NP surface. Finally, bacterial adhesion‐inhibition studies showed the inhibitory potential of mannose bearing gCs and gC‐GNPs. Overall, this study presents a versatile synthetic strategy to complex multivalent scaffolds based on the combination of solid‐phase synthesis, calixarenes and gold nanoparticles that can now also be used to present other ligands or functional groups for different applications.

## Supporting Information

The authors have cited additional references within the Supporting Information.^[^ ^33^ ^]^

## Conflict of Interest

The authors declare no conflict of interest.

## Supporting information



Supporting Information

## Data Availability

The data that support the findings of this study are available in the supplementary material of this article
