# Supplementary material for: A Modular Solid Phase Synthesis Approach for Glycocalix[4]Arene Derivatives and Their Multivalent Presentation on Ultrasmall Gold Nanoparticles
Source: Chemistry. 2025 Jun 27;31(40):e202500497. doi: 10.1002/chem.202500497 (PMC12272004; doi:10.1002/chem.202500497)
Supplement: Supplementary file 1 — Supporting Information [file CHEM-31-e202500497-s001.pdf]

Supporting Information

A Modular Solid Phase Synthesis Approach for Glycocalix[4]arene Derivatives and their Multivalent Presentation on Ultrasmall Gold Nanoparticles.

Alisa Kayser[a], Kai Klein[b], Daria Babushkina[a], Anna Sakse[a], Gisele Mouafo Kenne[a], Hajo Böschen[a], Ulla I.M. Gerling-Driessen[c], Monir Tabatabai[a], Matthias Epple[b] and Laura Hartmann[a][c]

[a] Department of Organic Chemistry and Macromolecular Chemistry, Heinrich Heine University Dusseldorf, Universitätsstraße 1, Dusseldorf, 40225, Germany.

[b] Inorganic Chemistry and Center for Nanointegration Duisburg-Essen (CENIDE), University of Duisburg Essen, Universitätsstraße 7, Essen 45141, Germany.

[c] Institute for Macromolecular Chemistry, University of Freiburg, Stefan-Meier Straße 31, Freiburg i.Br. 79104, Germany

Materials:

The following chemicals were used without further purification: Dimethylformamide (≥99.8%, Biosolve Chemicals) , dichloromethane (≥99.8%, Fisher Scientific), chloroform (≥99.8%, Fisher Chemical), tetrahydrofuran (≥99.9%, Sigma Aldrich), 1,4-dioxane (98%, Sigma Aldrich), N-methyl-2-pyrrolidone (≥99.0%, Acros Organics), methanol (≥99.8% VWR Chemicals), acetonitrile (HPLC grade, ≥99.9%, Honeywell), diethyl ether (≥99.8%, Honeywell), dimethyl sulfoxide (≥ 99%, Carl Roth), N_β-boc-N_α-Fmoc-L-2,3-diaminopropionic acid (>97%, TCI chemicals), 4-pentynoic acid (95%, Sigma Aldrich), TentaGel S RAM (Rapp Polymere), AG® 1-X8 (Bio-Rad Laboratories), benzotriazole-1-yloxytripyrrolidinophosphonium hexafluorophosphate (≥98%, Carbosynth limited), N,N-diisopropylethylamine (≥99.0%, Carl Roth) N-methyl morpholine (> 99.0% Tokio Chemical Industry), piperidine (99%, Acros Organics), triisopropyl silane (98 %, Sigma Aldrich), sodium methoxide (≥ 97%, Sigma Aldrich), tin(II) chloride dihydrate (≥98%, Carl Roth), sodium ascorbate (≥ 99 %, Carl Roth), copper(II) sulfate (>98%, Acros Organics), sodium diethyldithiocarbamate trihydrate (98%, Alfa Aesar), 4-*tert*-butylcalix[4]arene (≥98%, Biosynth Carbosynth), barium oxide (99.5%, abcr), barium hydroxide octahydrate (≥98%, Sigma Aldrich), 1-iodopropane (99%, Sigma Aldrich), sodium hydride (60% dispersion in mineral oil, Sigma Aldrich), ethyl 6-bromohexanoate (≥97%, Fluorochem), nitric acid (≥99%, Sigma Aldrich), acetic acid (≥99.8%, Sigma Aldrich), hydrochloric acid (37%, Fisher Chemical), trifluoroacetic acid (99.5 %, Acros Organics), potassium hydroxide (≥99.5%, Sigma Aldrich), tris(3-hydroxypropyl-triazoylmethyl) amine (96%, Sigma Aldrich), aminoguanidine hydrogen carbonate (98%, Alfa Aesar), Amberlite IR 120 (Sigma Aldrich), phenol (99%, Fisher Scientific), methyl α-D-mannopyranoside (≥99.0%, Sigma Aldrich), mannan from *Saccharomyces cerevisiae* (Sigma Aldrich), Bovine Serum Albumin (Sigma Life Science),

Milli-Q water was gained by a MicroPure water purification system from ThermoScientific.

Buffer and media: Phosphate-buffered saline (PBS, pH 7.4) was prepared using PBS buffer tablets from Carl Roth for 100 ml. For PBST 0,05 vol.-% Tween® 20 (polyoxyethylen-20-sorbitanmonolaurat, Carl Roth) was added to PBS buffer. For the Carbonate puffer (pH 9.4) 1.59 g sodium carbonate and 2.52 g sodium hydrogen carbonate was dissolved in 1L MQ water. LB-media was prepared by dissolving 25 g LB miller broth from Sigma Aldrich in 1 L MQ water. After sterilization 50,0 mg of ampicillin sodium salt (Sigma Aldrich) and 25,0 mg chloramphenicol (BioChemica, AppliChem) were added. The pH value was adjusted with 0.1 M NaOH using a pH-electrode by Mettler Toledo.

imidazole-1 sulfonyl azide hydrogen sulfate was synthesized according to a previously communicated protocol.[1]

The building blocks TDS ((triple-bond diethylenetriamine-succinic acid, 1-(fluorenyl)-3,11-dioxo-7- (pent-4-ynoyl)-2-oxa-4,7,10-triazatetra-decan-14-oic acid) and EDS (EDS (ethylene glycoldiamine-succinic acid 1-(9H-fluoren-9-yl)-3,14-dioxo-2,7,10-trioxa-4,13-diazaheptadecan-17-oic acid) were synthesized as previously reported.[2]

The synthesis of (2-azidoethyl)-2,3,4,6-tetra-O-acetyl-α-D-mannopyranoside was performed according to literature.[3] (2-azidoethyl)-2,3,4,6-tetra-O-acetyl-β-D-galactopyranoside was synthesized as reported previously.[4]

Instruments:

Reversed Phase – High Pressure Liquid Chromatography (RP-HPLC-MS):

Chromatography measurements were performed on an Agilent 1260 Infinity instrument coupled to a variable wavelength detector at an absorption wavelength of 214 nm and an Agilent Quadrupole mass spectrometer with an electrospray ionization (ESI) source (m/z range from 200-2000). A MZ-Aqua Perfect C18 column (3.0 x 50 nm, 3 µm) was used at 25° C. As mobile phase linear gradients of an eluent system A and B was used: A) H_20/ACN (95/5 vol.%) + 0.1 vol.-% formic acid; B) H_20/ACN (5/95 vol.%) + 0.1 vol.% formic acid). The flow rate was 0.4 ml/min.

Preparative RP-HPLC:

Chromatographic purifications were done on an Agilent 1200 Series instrument with variable wavelength detector at 214 nm and a fraction collector was used. A UG80 C18 RP column (20 x 250 nm, 5 µM) from Shiseido was used at 25° C. A flow rate of 10 ml/min was applied. For all samples an eluent system A and B was used in linear gradients: A) H_2O + 0.1 vol.% formic acid; B) ACN + 0.1 vol.-% formic acid.

Matrix Assisted Laser Desorption Ionization – Time of Flight – Mass Spectrometry (MALDI-TOF-MS):

The MALDI-TOF mass spectra were measured on a Bruker UltrafleXtreme instrument from Bruker Daltonics. The measurements were performed using 2,5-dihydroxybenzoic acid (DHB) or dithranol (DIT) in combination with sodium trifluoroacetate as a matrix in linear or reflector mode.

^{1}H-Nuclear Magnetic Resonance (^{1}H-NMR):

^{1}H-NMR spectra were either recorded on a Bruker Avance III-300 (300 MHz) or 600 (600 MHz). The chemical shifts are reported in delta (δ) expressed in parts per million (ppm). The residual, non-deuterated solvent (δ 3.31 ppm for MeOH-*d4* or δ 2.50 ppm for DMSO-*d6*) were used as an internal reference. The nanoparticles were dispersed in a H_2O/D_2O mixture (90/10 vol.-%) and water signal suppression was used.

Fluorescence Spectroscopy:

All fluorescence emission measurements were acquired on a CLARIOstar® microtiter plate reader from BMG LABTECH at 485/535 nm at ambient temperature. All measurements were performed with black polystyrene 96-wells microtiter plates from Greiner. The data were evaluated with the BMG Mars software.

Fluorescent Microscopy:

The fluorescence microscopy measurements were performed on an inverted microscope IX73 from Olympus with an Olympus 60 x NA 1.35 oil-immersion objective and a CMOS camera (UI-3360CP-M-GL). For the measurement µ-slides 18 wells with glass bottom from ibidi were used. As a light source a collimated LED (530 nm, blue light) from Thorlabs was used.

Atomic Absorption Spectroscopy (AAS):

AAS was done with an Electron M-series spectrometer from ThermoScientific after dissolving the gold nanoparticles in *aqua regia*.

Disc centrifugal sedimentation (DCS):

DCS was done with a DC24000 instrument from CPS. Thereby, the disc was accelerated to 24000 rpm. A gradient of sucrose solution was injected starting 1.6 mL of 24 wt.-% sucrose in water, the gradient increased in steps of 0.2 mL to 1.6 mL of 8 wt.-% sucrose in water. The gradient solution was capped with 0.5 mL of dodecane to avoid evaporation. The instrument was calibrated with a dispersion of stabilized PVC particles with a defined hydrodynamic diameter of 483 nm.

Freeze-dryer:

The final products were freeze-dried with an Alpha 1-4 LD plus instrument from Martin Christ set to -50° C at 0.1 mbar.

Solid phase synthesis protocol:

Coupling and Fmoc-deprotection procedure:

All solid phase reactions were performed on TentaGel S RAM resin in batch sizes up to 0.15 mM in polypropylene syringes with integrated frits. Prior to use, the resin was swollen in DCM for 30 mins followed by washing with DMF five times. The reaction conditions for the coupling of different building blocks are given in the table below (table S1) and differ prior to and subsequent the introduction of CBB2. The coupling steps prior to coupling of CBB1 was performed according to a previously established coupling protocol using 5 eq. PyBOP and 10 eq. DIPEA as coupling reagents.[2b] For the coupling of EDS to CBB1, reaction conditions were optimized. Using DIPEA as a base resulted in relative purities of 83-89%, while N-methyl morpholine yielded purities of 86-95%, depending on the prior coupling sequence. As additional by-product are formed using DIPEA, *N*-methyl morpholine is used as a base as standard procedure. After coupling, the resin was washed 10 times with DMF and DCM. Fmoc deprotection was performed with 20 vol.-% piperidine in DMF for 30 min (fresh solution added after 20 min). The resin was washed 10 times with DMF before the next coupling step.

Table S1. Reaction conditions for coupling steps prior to and subsequent the integration of the calix[4]arene building block CBB1

	Building block	Eq. of building block	Coupling agents	Reaction time
Prior CBB1	EDS/TDS/Dap/ pentynoic acid	5	5 Eq. PyBOP + 10 Eq. DIPEA	1h
-	CBB1	3	5 Eq. PyBOP + 10 Eq. DIPEA	5h
Subsequent CBB1	EDS/TDS/ pentynoic acid	5/amine	5. Eq. PyBOP/amine + 10 Eq. *N*-methyl morpholine/amine	1h

Boc group removal:

The Boc group in the side chain of Dap was removed using 4M HCl in dioxan solution. The resin was first washed five times with dioxan before the HCl solution was added to the syringe and shaken vigorously for 10 min. The resin was then washed three times with dioxan and the procedure was repeated for a further 20 min. For neutralization, the resin was shaken (2x10 min) in 10 vol% DIPEA in DCM. The resin was then washed three times with approximately 2 ml dioxan and DCM and finally ten times with 2 ml DMF. This was followed by a coupling step according to the protocol described above.

Reduction of Aryl-nitro groups:

The nitro groups at the upper rim of CBB1 were reduced to amines by treating the resin with 25 eq. tin(II) chloride dihydrate in NMP (3 ml) for 24 hours at 30° C. To remove excess of tin salt, the resin was washed thoroughly ten times with NMP, methanol, DCM and DMF.

Copper(I)-catalyzed azide-alkyne cycloaddition (CuAAC):

For the cycloaddition 2eq. of the carbohydrate azide was dissolved in 1 ml DMF. In addition, 20 mol-% relative to the azide component of sodium ascorbate as well as copper(II) sulfate was dissolved separately in 0,1 mL of MQ water each. The solutions were then added to the syringe in the mentioned order and shaken overnight in the dark. By washing the resin

alternately with a 0,23 M solution of sodium diethyldithiocarbamate in DMF/water and DMF the excess copper is removed, which can be monitored by color change.

Deacetylation carbohydrate residues:

Approximately 2 ml of a 0.2 M sodium methanolate solution in methanol was added to the syringe and shaken vigorously for 30 mins. Subsequently, the resin was washed ten times with methanol and DMF.

Cleavage from the solid phase:

A solution of TFA/TIPS/DCM (95/2.5/2.5 vol.-%) was used to cleave the final glycocalix[4]arene from the solid support. The resin was treated with the cleavage solution for 1h and subsequently the product was precipitated in ice-cold diethyl ether. After centrifugation at 4000 rpm the supernatant was decanted, and the remaining precipitate was dissolved in water or water/DMSO (for water-insoluble derivative C4 and C5) and finally lyophilized.
To monitor the reactions on the solid support, small amounts of the resin were separated and treated according to the cleavage conditions described above (referred to as micro cleavage). The obtained precipitate was then analyzed via RP-HPLC-MS.

TFA anion exchange:

For the anion exchange of the water-soluble derivatives (C1-C3 and C56-C7) an AG® 1-X8 resin was used. The resin was first washed three times with 1,6 M aqueous acetic acid followed by three times with 0,16 M acetic acid before use. The exchange was performed for two hours.

Sulfuric acid phenol method:

The total concentration of carbohydrates in each sample was determined by the established sulfuric acid phenol method.[5] Here, the reaction is performed in polystyrene microtiter plates, as less sample volume is required. D-mannose in nanoparticle dispersion was used for standard curves. D-mannose was used in a concentration range from 320 to 20 µM, while the nanoparticle concentration was kept constant at 41.11 µM (C4-GNPs, C5-GNPs and m-GNPs) or 6.85 µM (C6-GNPs, C7-GNPs). This procedure was performed because the ultra-small nanoparticles cannot be properly removed from the carbohydrate sample, causing background noise that must be considered. To 35 µL of the sample, 35 µL of a 5 w.-% phenol solution in MQ water was added followed by 180 µL of concentrated sulfuric acid and mixed vigorously with a pipette. The mixture was then incubated for 30 min at 30°C. The absorbance of each sample was detected at 420 nm. All measurements were performed in triplicate. As a reference, the gold nanoparticles were treated analogously before functionalization. By comparing the sample with the standard curve, the carbohydrate concentration was determined.

Bacterial adhesion-inhibition assay:

For the bacterial adhesion inhibition studies, a E. Coli strain pPKL1162, kindly provided by the Lindhorst group from the University of Kiel, Germany, was used. The assay was performed according to literature protocols with slight modifications.[6] The bacteria were cultured from a frozen stock (LB media + AMP + CAM) over night at 37° C in LB media. The bacteria were then washed three times with PBS buffer and subsequently diluted with PBS buffer to obtain a bacterial dispersion with OD(600 nm) = 0.4. The polystyrene microtiter plates were mannan coated with 120 µL of mannan from *Saccharomyces cerevisiae* overnight in carbonate buffer (pH = 9.4) at 37° C. The plates were washed three times with PBST buffer (150 µL, PBS + 0,05% Tween) and then blocked with 120 µL of 0,1% BSA in PBS solution for two hours. Subsequently, the plates were washed three times with PBST (150 µL). Afterwards, the samples were applied to the microtiter plates in a serial dilution in PBS buffer (50 µL) and incubated with bacterial dispersion (50 µL) for 45 min at 45

min. Then, the plates were washed with PBS buffer (100 µL) and the wells were filled with 100 µL PBS. The fluorescence read out was measured at 485/535nm.

Synthesis of nanoparticles and functionalization by CuAAC

The gold nanoparticles Au-GSH were prepared according to an adapted Brust-Schiffrin protocol as previously reported.[1] Subsequently, the azidation of the nanoparticles was performed as reported, except that the equivalents of the imidazole-1 sulfonyl azide hydrogen sulfate were reduced to 15 eq. compared to 50 eq. in the original protocol, with respect to glutathione. CuAAC of the alkyne-functionalized glycocalix[4]arenes **C4-C7** to the AuGSH-N_3 nanoparticles was carried out as follows. The nanoparticle sample containing 7.7 µmol azido groups (68 nmol, 3.15 mg Au) was dispersed in 3 mL of water. Then the glycocalix[4]arenes **C4-C7** were dissolved in 1 mL of water or DMSO (0,4 eq., 3.08 µmol) and added to the nanoparticle dispersion. Subsequently, 630 µL of an aqueous solution of $CuSO_4$, THPTA (50 µmol) and aminoguanidine hydrogen carbonate (10 µmol), previously prepared in 10 mL of water, were added to the reaction mixture. Finally, sodium ascorbate in water (3.15 µmol, 0.63 mg in 100 µL water) was added to start the reaction. The mixture was stirred for 14 h before water was added to give an overall volume of 20 mL, followed by purification by spin filtration (4x) (Amicon spin filters).

The number of carbohydrate molecules was computed as reported earlier, assuming an average diameter of the solid core of a spherical gold nanoparticle of 2 nm, by dividing the concentration of carbohydrate molecules (determined by the sulfuric acid phenol test)[5] in the dispersion by the concentration of gold nanoparticles in the dispersion (determined by AAS).[7]

Analytical Data

α-D-propargyl-mannopyranoside:

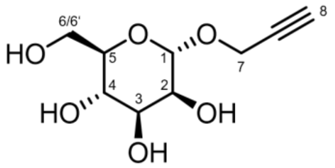

2,3,4,6-Tetra-*O*-acetyl-α-D-propargyl-mannopyranoside was synthesized according to a previously reported protocol.[8] For deacetylation the carbohydrate was dissolved in a 2 mg/mL solution of sodium methoxide in methanol and let stir for 50 minutes. The reaction was stopped by adding Amberlite IR120 Hydrogen Form until the pH is neutral. Subsequently, the resin was filtered off and the product was obtained after concentration under reduced pressure.

^{1}H-NMR (300 MHz, D_2O): δ 5.04 (d, ^3J=1.9 Hz, 1H, 1), 4.34 (dd, ^2J=5.7 Hz, ^4J=2.5 Hz, 2H, 7), 3.96 (dd, ^3J = 1.9, 3.4 Hz, 1H, 2), 3.9 (dd, ^3J = 1.8, 12.2 Hz, 1H, 4), 3.84-3.65 (m, 4H, 3, 5, 6, 6'), 2.93 (t, ^4J=2.5 Hz, 1H, 8) ppm.

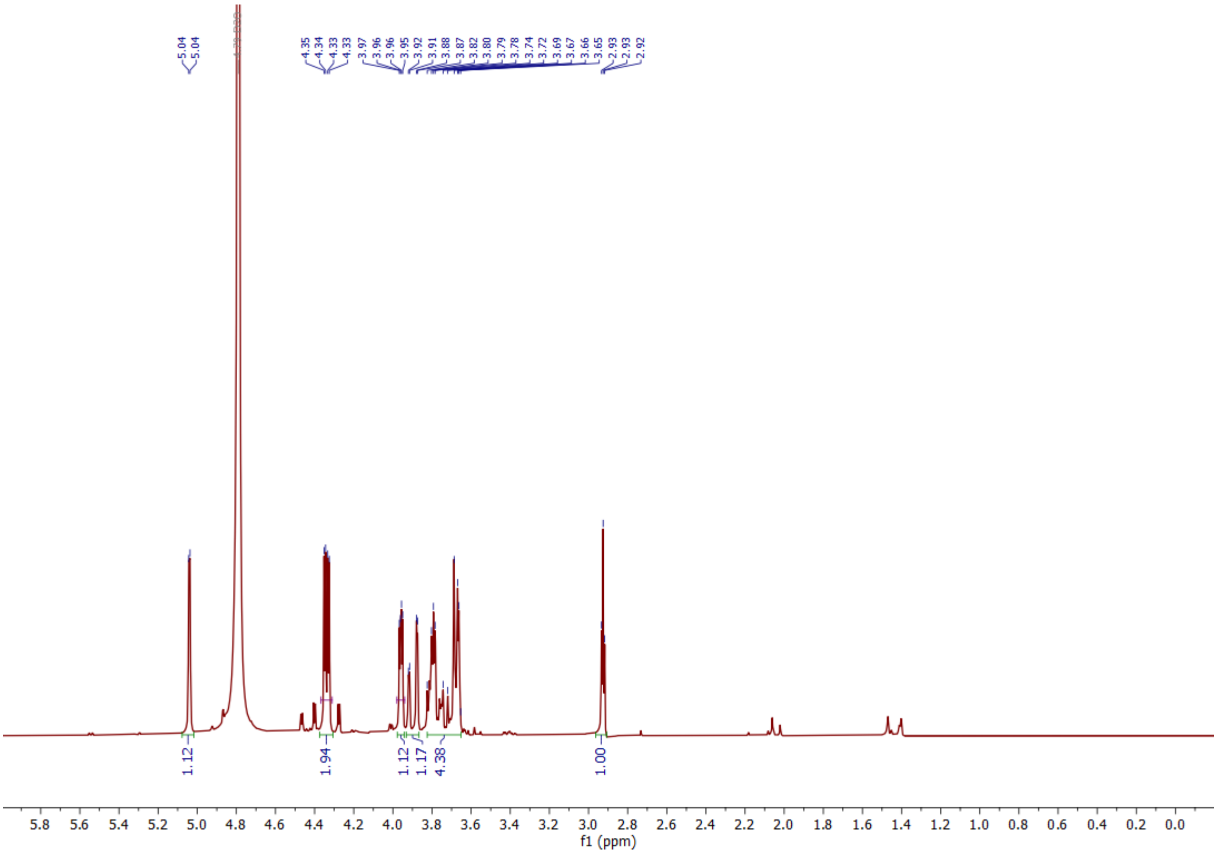

Figure S1. ^{1}H-NMR spectrum (300MHz) of α-D-propargyl-mannopyranoside in D_2O.

Calix[4]arene building block CBB1:

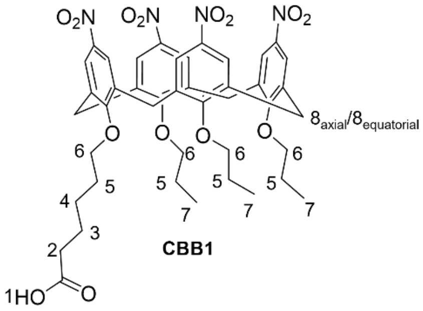

CBB1

The building block was prepared as previously reported.[9]

^{1}H-NMR (300 MHz, DMSO-*d6*): δ 12.04 (s, 1H, 1), 7.65 (s, 8H, Aryl-H), 4.37 (d, ^{2}J= 12.1 Hz, 4H, 8_{axial}), 3.95 (m, 8H, 6), 3.69 (d, ^{2}J=12.1 Hz, 4H, $8_{equatorial}$), 2.24 (t, ^{3}J = 7.2, 2H, 2), 1.85 (m, 8H, 5), 1.58 (p, ^{3}J=7.1 Hz, 2H, 3), 1.41 (m, 2H, 4), 1.05-0.90 (m, 9H, 7) ppm.

MALDI-TOF-MS: m/z calculated for $C_{43}H_{48}N_4O_{14}$: 867.32 $[M+Na]^+$, found: 867.36 $[M+Na]^+$

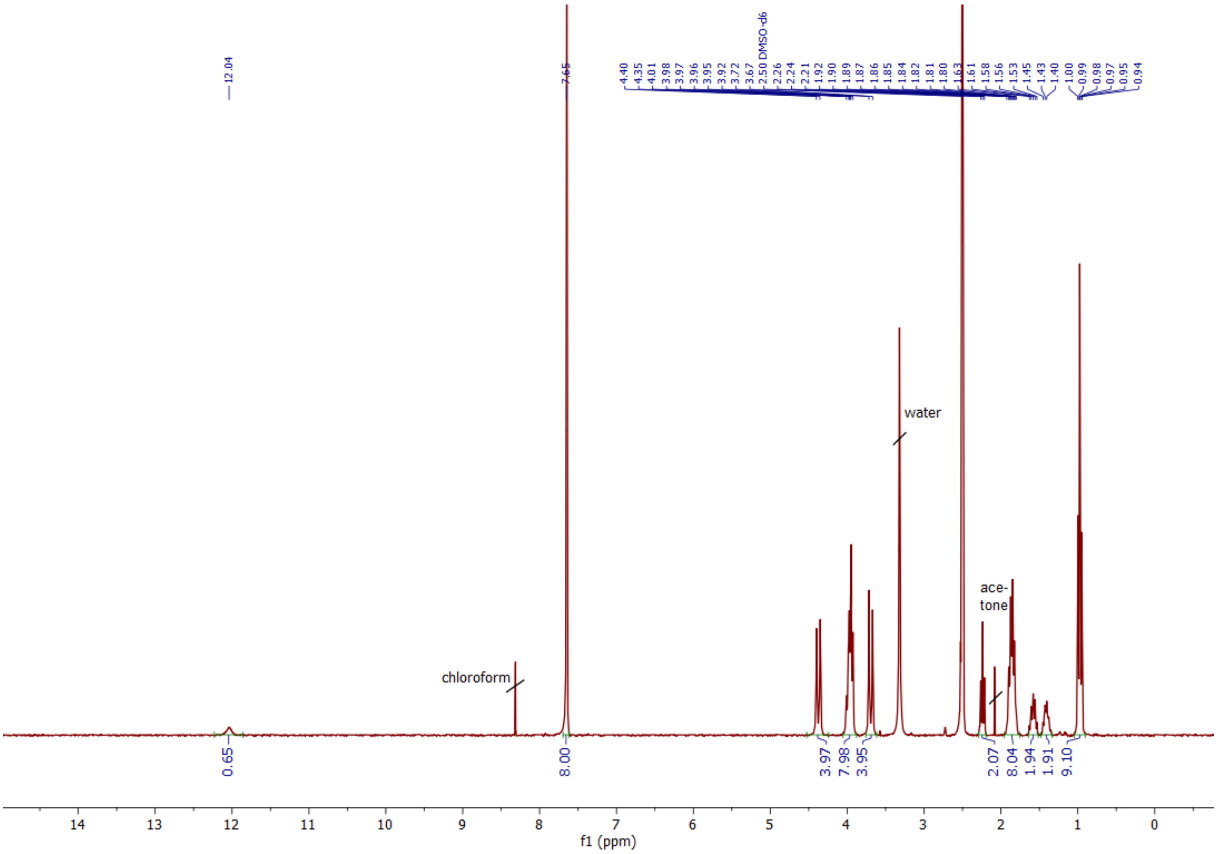

Figure S2. ^{1}H-NMR spectrum (300MHz) of CBB1 in DMSO-*d6*.

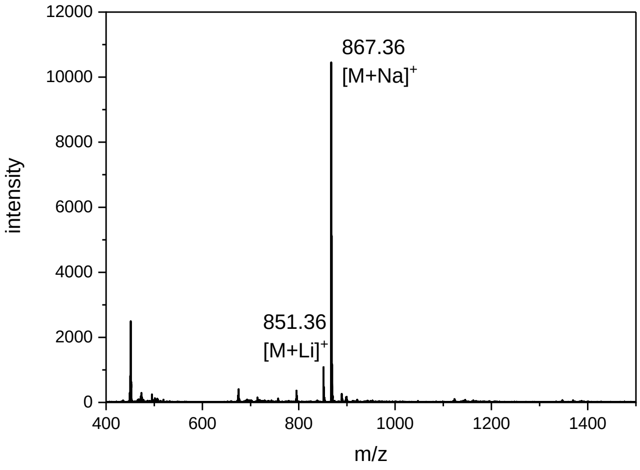

Figure S3: MALDI-TOF-MS spectrum of CBB1.

Compound C1:

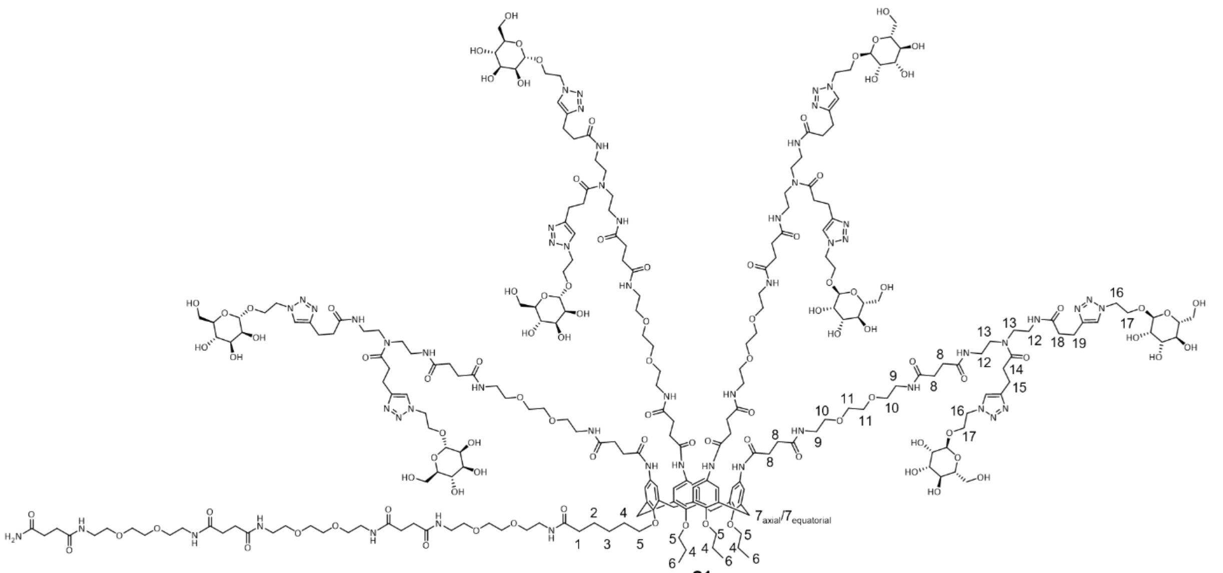

C1

Molecular Weight: 5711.19

Compound **C1** was purified by preparative HPLC (73% A to 67% A in 13 min) and obtained in a yield of 10%. The relative purity of 90% was determined by integration of the UV signals of the RP-HPLC run at 214 nm. MS analysis suggests that the by-product observed, that could not be separated by preparative purification, is due to EDS-TDS-(man)-pentynoic acid(man) deletion. Additionally, ^{1}H-NMR analysis confirms this.

ESI-MS: m/z calculated for $C_{249}H_{395}N_{55}O_{97}$: 1427.94 $[M+4H]^{4+}$, 1142.55 $[M+5H]^{5+}$, 952.3 $[M+6H]^{6+}$, 816.4 $[M+7H]^{7+}$, 714.47 $[M+8H]^{8+}$, found: 1428.7 $[M+4H]^{4+}$,1143.2 $[M+5H]^{5+}$, 952.8 $[M+6H]^{6+}$, 816.75 $[M+7H]^{7+}$

MALDI-TOF-MS: m/z calculated for $C_{249}H_{395}N_{55}O_{97}$: 5734.19 $[M+Na]^{+}$, found: 5734.0

^{1}H-NMR (600 MHz, MeOH-*d₄*/D_2O): δ 8.1-7.94 (m, 1H, NH), 7.98-7.71 (m, 8H, Triazole-H), 6.95-6.77 (bs, 8H, Aryl-H), 4.72 (m, 4.71-4.72, 8H, $CH_{mannose}$), 4.45-4.41 (m, 4H, 7_{axial}), 4.14-4.04 (m, 8H, $CH_{mannose}$), 3.93-3.33 (m, 185H, $CH_{mannose}$, 5, 9, 10, 11, 12, 13, 17 overlaps with solvent peak) 3.18-3.06 (m, 12H, $CH_{mannose}$, $7_{equatorial}$) 3.03-2.92 (m, 16H, 15, 19), 2.84-2.73 (m, 8H,14), 2.62-2.41 (m, 54H, 8, 18) 2.25 (t, ^{3}J= 7.4 Hz , 2H, 1), 2.00-1.88 (m, 8H, 4), 1.71 (p, ^{3}J= 7.7 Hz, 2H, 2), 1.51-1.43 (m, 2H, 3), 1.06-0.97 (m, 9H, 6) ppm.

The signals of protons 16 overlap with water peak at 4.59 ppm. Signals from unidentified impurities can be found at 1.29-1.34 ppm.

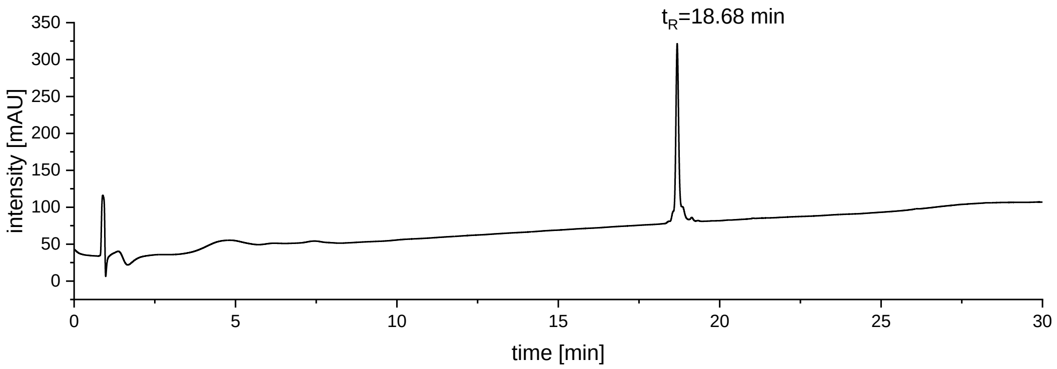

Figure S4. RP-HPLC chromatogram (100% A to 50% A in 30 min at 25° C) of compound C1.

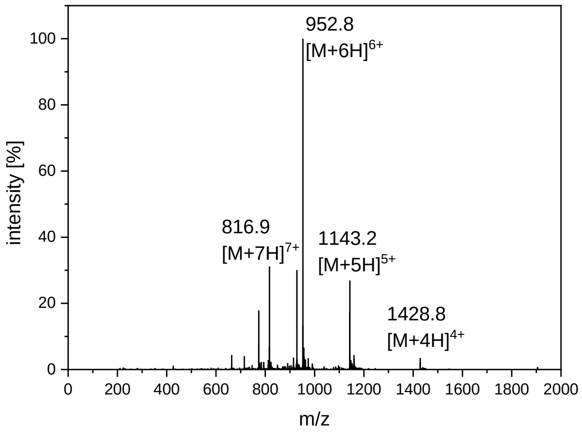

Figure S5. ESI-MS spectrum at t_R = 18.68 min (100% A to 50% A in 30 min at 25° C) of compound C1.

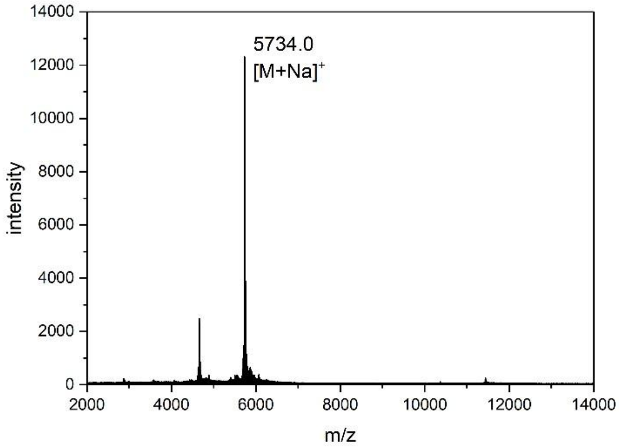

Figure S6. MALD-TOF-MS spectrum of compound C1.

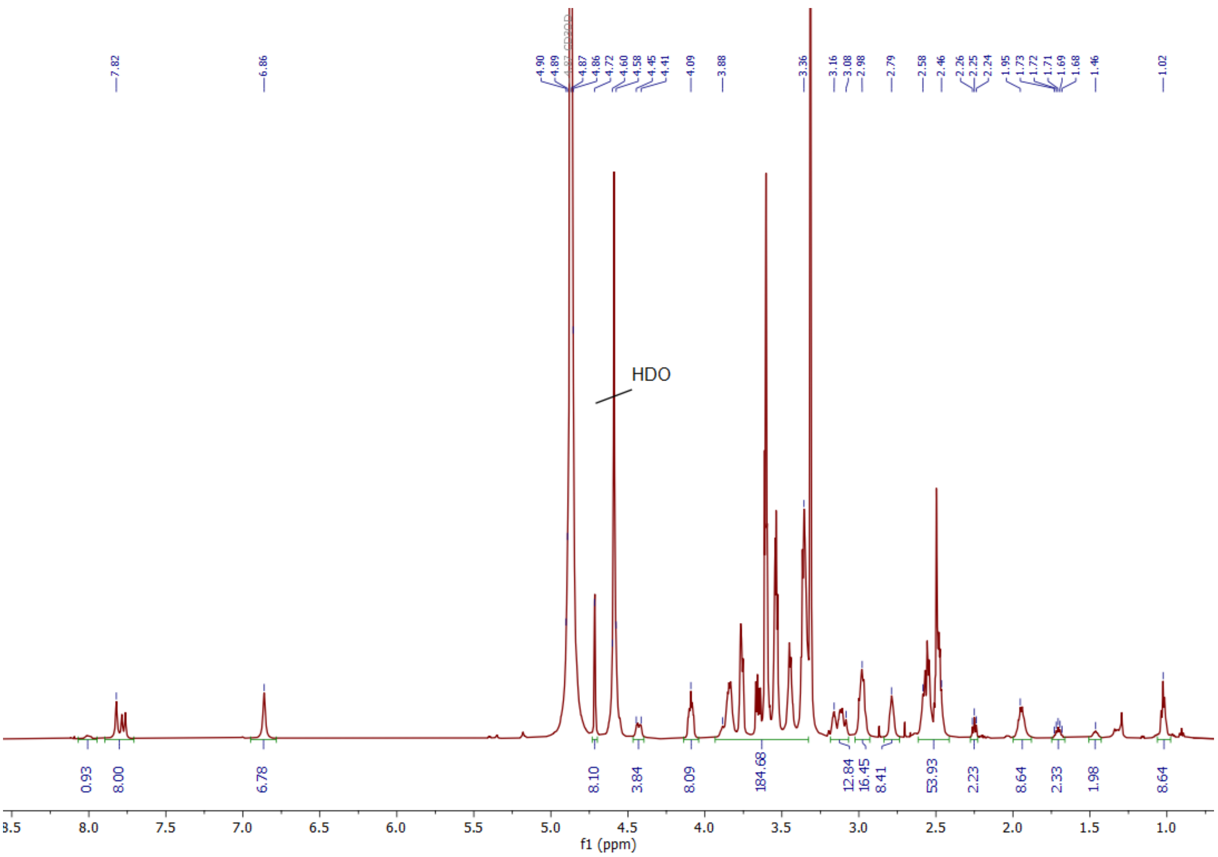

Figure S7: ^{1}H-NMR spectrum (600MHz) of compound C1 in MeOH-*d4*/D_2O.

Compound C2:

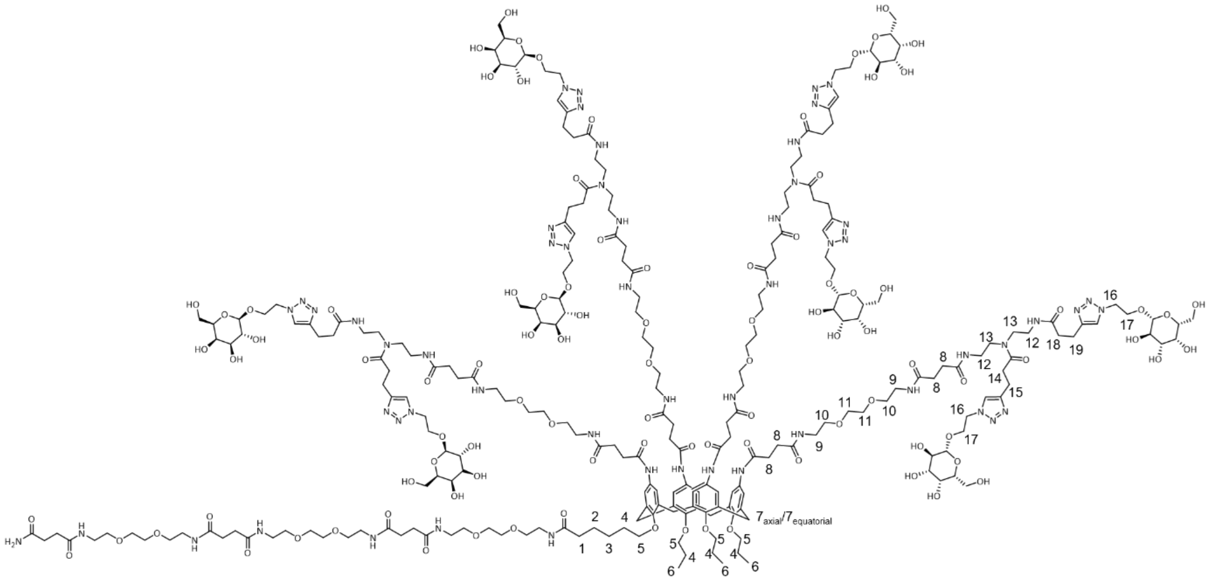

C2
Molecular Weight: 5711,19

Compound **C2** was purified by preparative HPLC (73% A to 67% A in 13 min) and obtained in a yield of 9%. The relative purity of 90% was determined by integration of the UV signals of the RP-HPLC run at 214 nm. MS analysis suggests that the by-product observed, that could not be separated by preparative purification, is due to EDS-TDS-(man)-pentynoic acid(man) deletion. Additionally, ^{1}H-NMR analysis confirms this.

ESI-MS: m/z calculated for $C_{249}H_{395}N_{55}O_{97}$: 1427.94 $[M+4H]^{4+}$, 1142.55 $[M+5H]^{5+}$, 952.3 $[M+6H]^{6+}$, 816.4 $[M+7H]^{7+}$, 714.47 $[M+8H]^{8+}$, found: 1428.6 $[M+4H]^{4+}$, 1143.2 $[M+5H]^{5+}$, 952.7 $[M+6H]^{6+}$, 816.7 $[M+7H]^{7+}$

MALDI-TOF-MS: m/z calculated for $C_{249}H_{395}N_{55}O_{97}$: 5734.19 $[M+Na]^{+}$, found: 5734.3

^{1}H-NMR (600 MHz, MeOH-d_4/D_2O): δ 8.48 (bs, 0.5H, -NH-), 8.07-7.78 (m, 8H, Triazole-H), 6.89 (bs, 6H, Aryl-H), 4.5-4.4 (m, 4H, 7_{axial}), 4.36-4.14 (m, 16H, $CH_{galactose}$), 4.06-3.33 (m, 182H, $CH_{galactose}$, 5, 9, 10, 11, 12, 13, 17, overlaps with solvent peak), 3.11 (d, 4H, ^{2}J = 13.0, $7_{equatorial}$) 3.05-2.99 (m, 16H, 15, 19), 2.88-2.72 (m, 8H,14), 2.66-2.42 (m, 53H, 8, 18) 2.26 (t, ^{3}J = 7.5 Hz , 2H, 1), 2.01-1.87 (m, 8H, 4), 1.72 (p, ^{3}J = 7.7 Hz, 2H, 2), 1.52-1.43 (m, 2H, 3), 1.07-1.00 (m, 7H, 6) ppm. The signals of protons 16 overlap with water peak at 4.59 ppm. Signals from unidentified impurities can be found at 1.28-1.38, 2.67, 2.71 and 2.88 ppm.

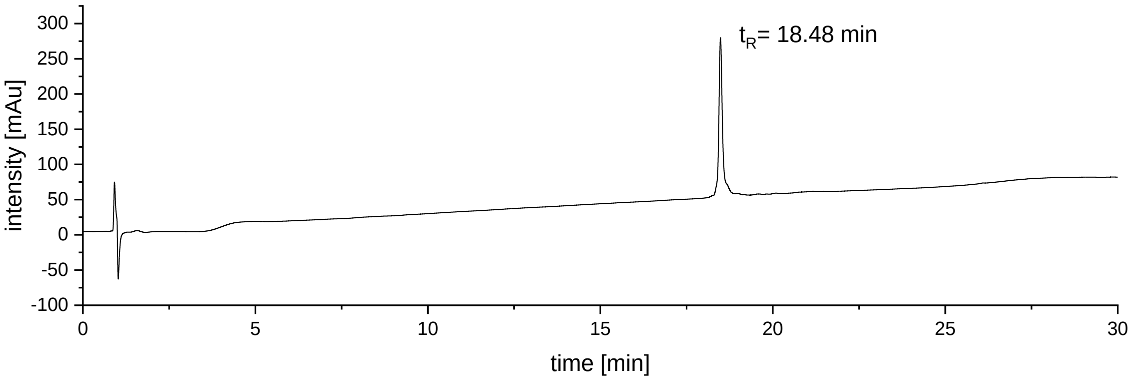

Figure S8. RP-HPLC chromatogram (100% A to 50% A in 30 min at 25° C) of compound C2.

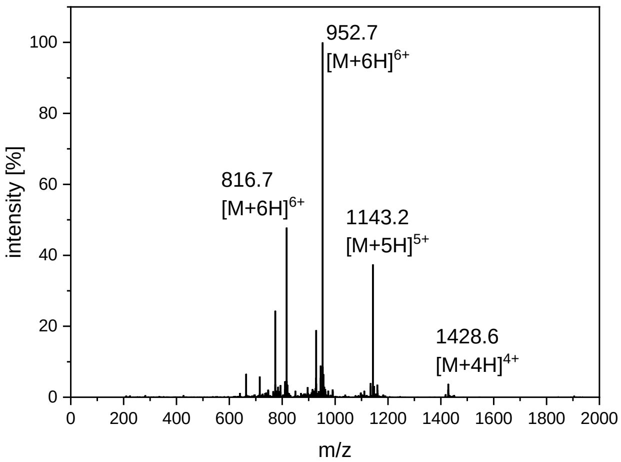

Figure S9. ESI-MS spectrum at t_R = 18.48 min (100% A to 50% A in 30 min at 25° C) of compound C2.

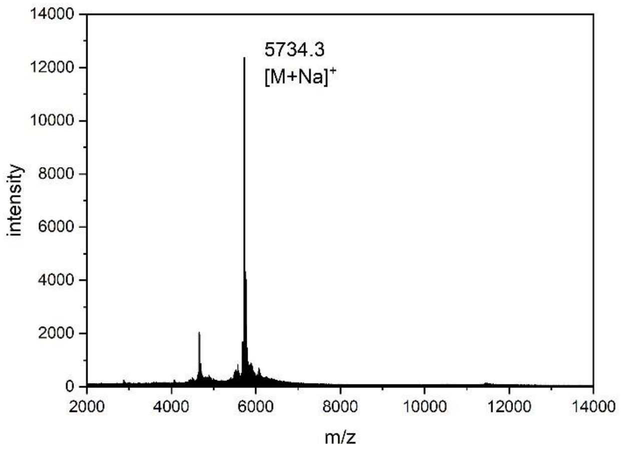

Figure S10. MALDI-TOF-MS spectra of compound C2.

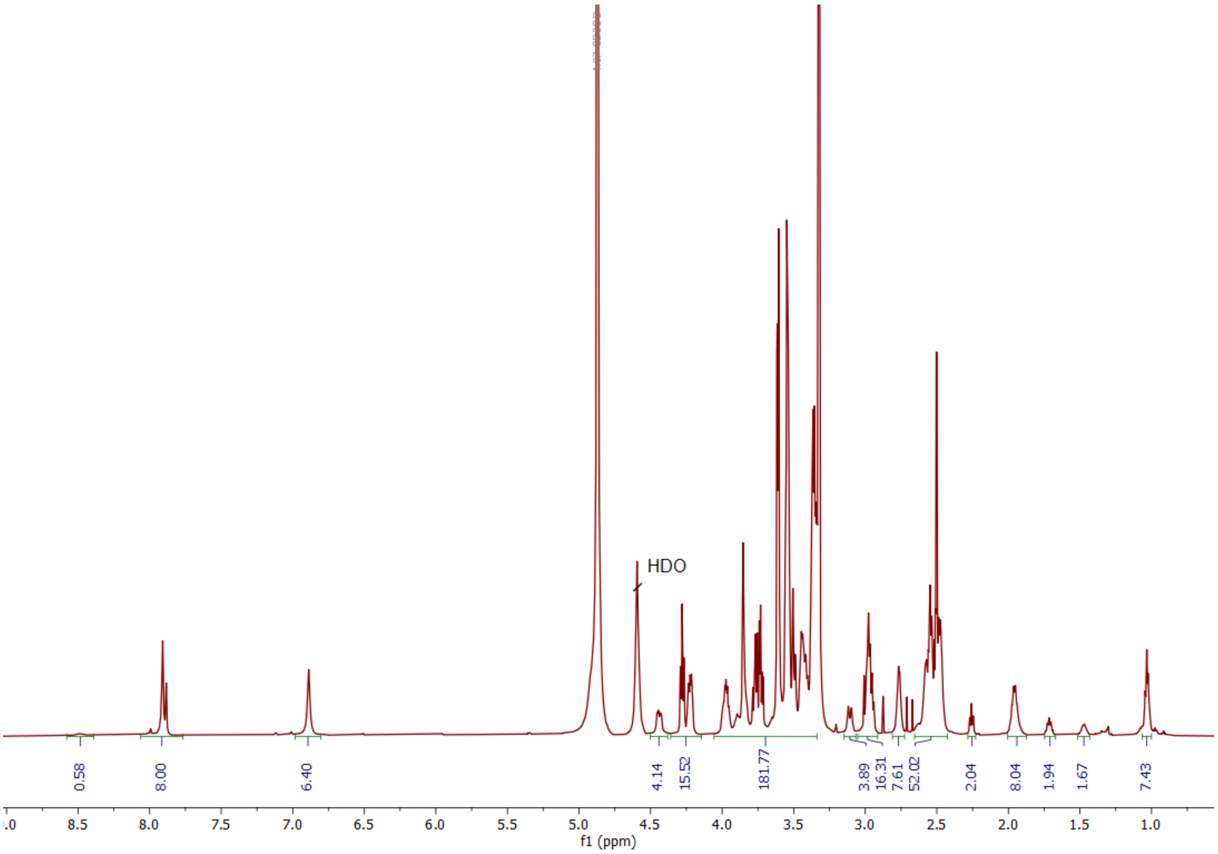

Figure S11. ^{1}H-NMR spectrum (600MHz) of compound C2 in MeOH-*d4*/D_2O. Phase correction was performed.

Compound C3:

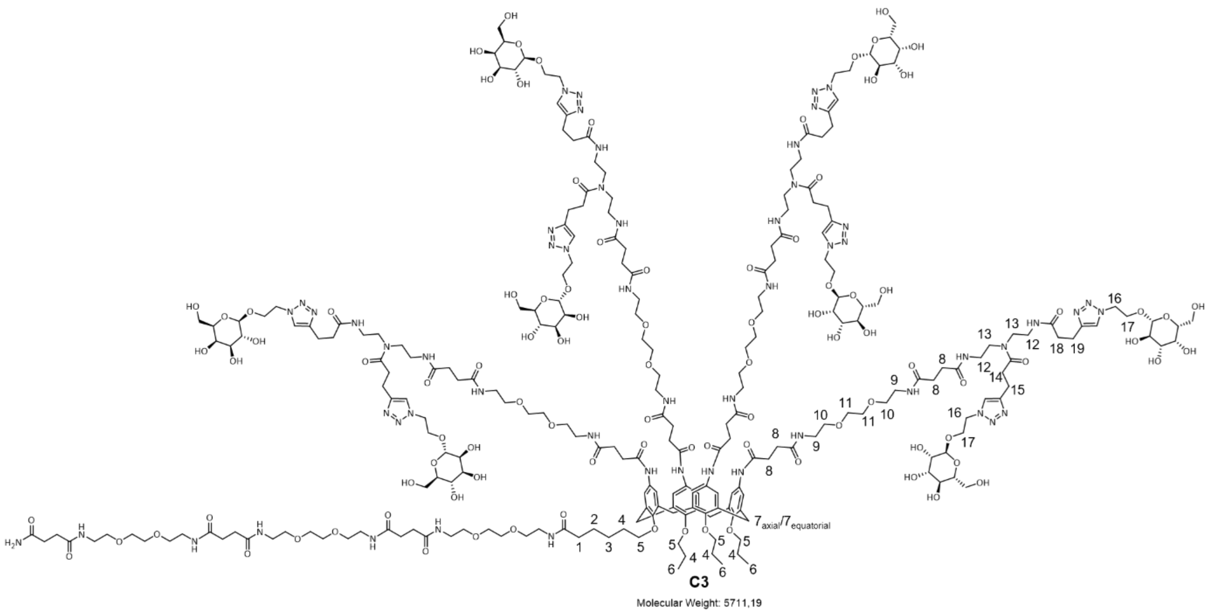

Compound **C3** was purified by preparative HPLC (73% A to 67% A in 13 min) and obtained in a yield of 9%. The relative purity of 95% was determined integration of the UV signals of the RP-HPLC run at 214 nm. MS analysis suggests that the by-product observed, that could not be separated by preparative purification, is due to EDS-TDS-(man)-pentynoic acid(man) deletion. Additionally, ^{1}H-NMR analysis confirms this.

ESI-MS: m/z calculated for $C_{249}H_{395}N_{55}O_{97}$: 1427.94 $[M+4H]^{4+}$, 1142.55 $[M+5H]^{5+}$, 952.3 $[M+6H]^{6+}$, 816.4 $[M+7H]^{7+}$, 714.47 $[M+8H]^{8+}$, found: 1428.6 $[M+4H]^{4+}$, 1143.1 $[M+5H]^{5+}$, 952.8 $[M+6H]^{6+}$, 816.8 $[M+7H]^{7+}$

MALDI-TOF-MS: m/z calculated for $C_{249}H_{395}N_{55}O_{97}$: 5734.19 $[M+Na]^{+}$, found: 5734.0

^{1}H-NMR (600 MHz, MeOH-*d₄*/D$_2$O): δ 8.06-7.71 (m, 8H, Triazole-H), 6.88 (s, 7H, Aryl-H), 4.74-4.70(m, 4H, $CH_{mannose}$), 4.65-4.54 (m overlap with water peak, 16) 4.5-4.39 (m, 4H, 7_{axial}), 4.33-4.29 (m, 4H, $CH_{galactose}$), 4.29-4.18 (m, 4H, $CH_{galactose}$), 4.13-4.06 (m, 4H, $CH_{mannose}$), 4.02-3.95 (m, 4H, $CH_{galactose}$), 3.93-3.33 (m, 181H, $CH_{mannose}$, $CH_{galactose}$, 5, 9, 10, 11, 12, 13, 17, overlaps with solvent peak), 3.16-3.07 (m, 8H, $7_{equatorial}$, $CH_{mannose}$,), 2.90-3.04-2.89 (m, 16H, 15, 19), 2.84-2.74 (m, 8H,14), 2.65-2.36 (m, 55H, 8, 18) 2.26 (t, ^{3}J= 7.5 Hz , 2H, 1), 2.00-1.9 (m, 8H, 4), 1.72 (p, ^{3}J= 7.8 Hz, 2H, 2), 1.52-1.43(m, 2H, 3), 1.07-1.0 (m, 8H, 6) ppm.
Signals from unidentified impurities can be found at 1.28-1.38, 2.67, 2.71 and 2.88 ppm.

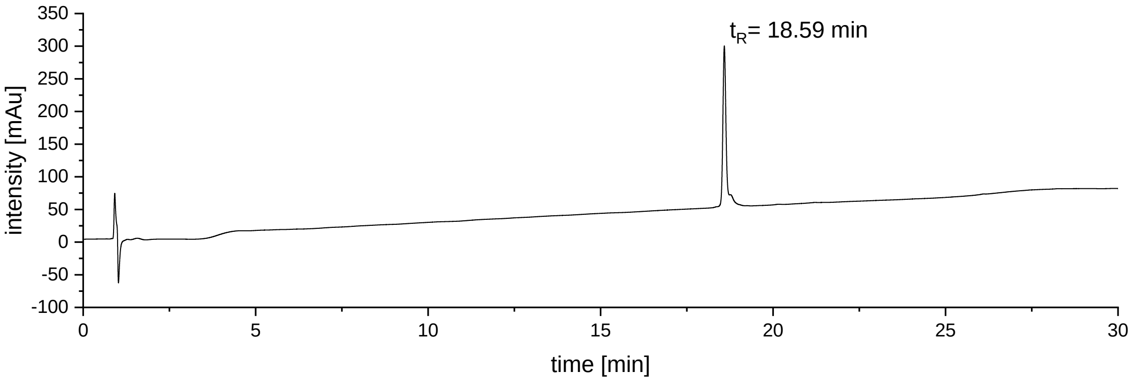

Figure S12. RP-HPLC chromatogram (100% A to 50% A in 30 min at 25° C) of compound C3.

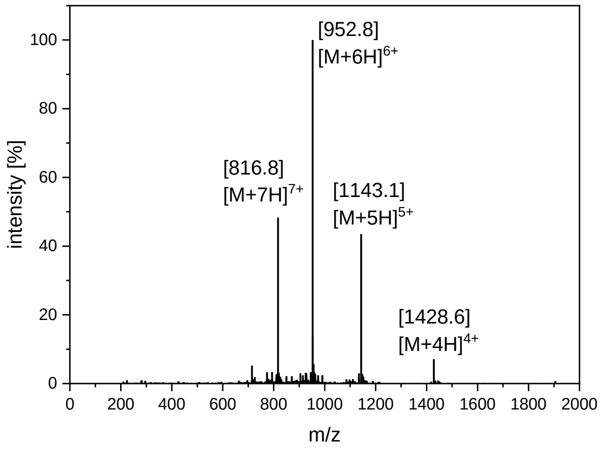

Figure S13. ESI-MS spectrum at t_R = 18.59 min (100% A to 50% A in 30 min at 25° C) of compound C3.

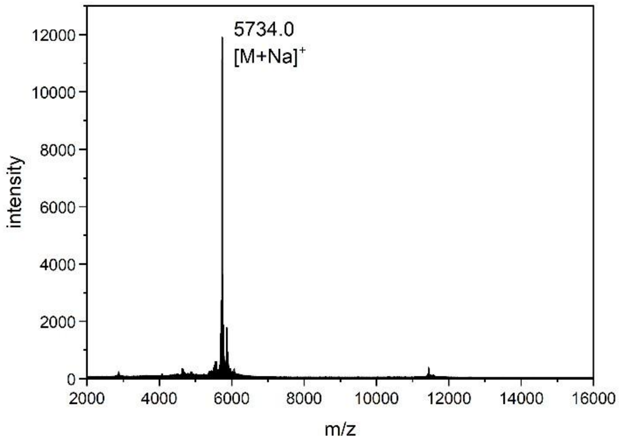

Figure S14. MALDI-TOF-MS spectrum of compound C4.

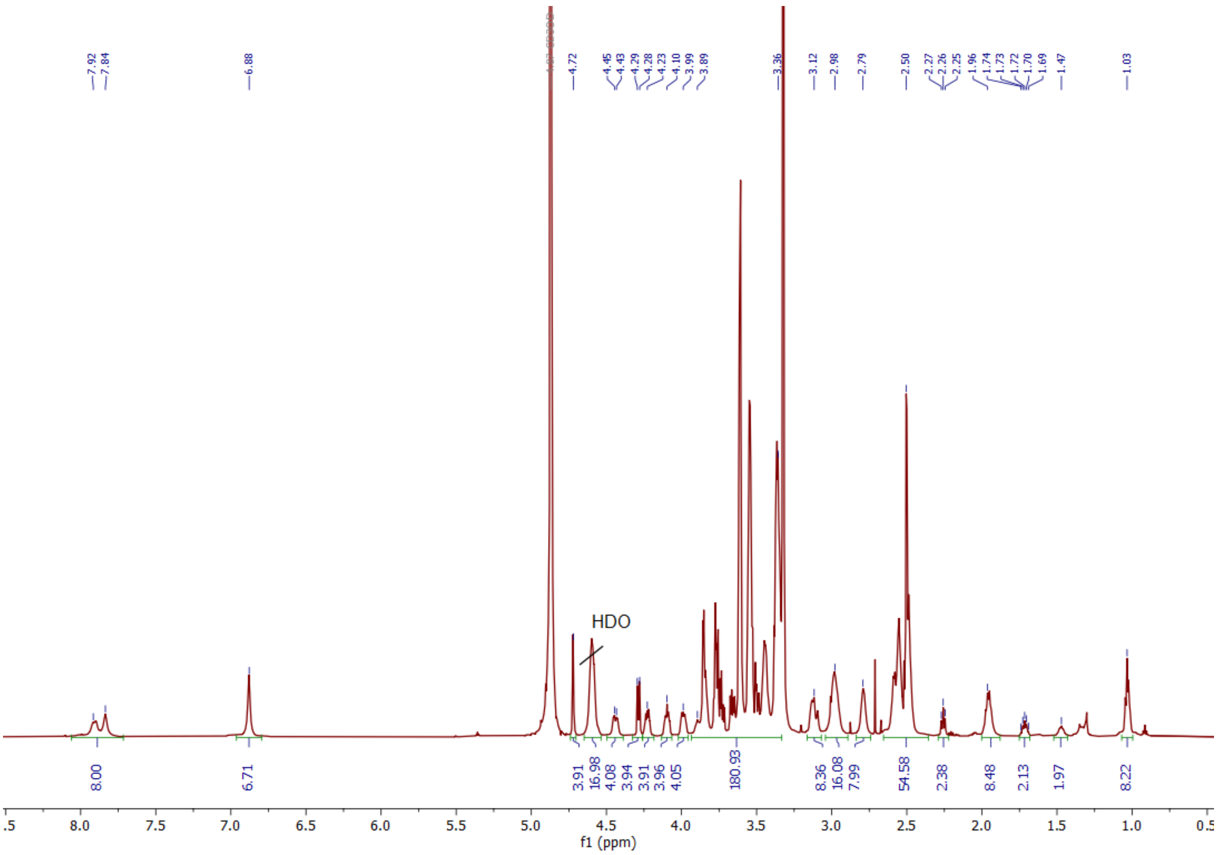

Figure S15. ^{1}H-NMR spectrum (600MHz) of compound C3 in MeOH-*d4*/D_2O.

Compound C4:

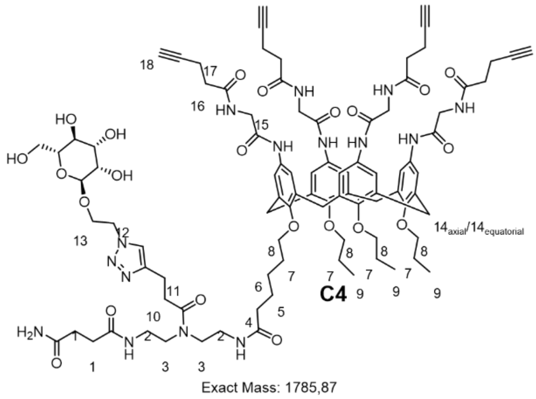

Compound **C4** was purified by preparative HPLC (60% A to 40% A in 15 min) and obtained in a yield of 45%. The relative purity of 89% was determined by integration of the UV signals of the RP-HPLC run at 214 nm.

ESI-MS: m/z calculated for $C_{92}H_{119}N_{15}O_{22}$: 1786.87 $[M+H]^{1+}$, 893.86 $[M+2H]^{2+}$, found: 1786.85 $[M+H]^{+}$, 894.05 $[M+2H]^{2+}$

MADLI-TOF-MS: m/z calculated for $C_{92}H_{119}N_{15}O_{22}$: 1786.87 $[M+H]^{+}$,1808.87 $[M+Na]^{+}$, found: 1810.30 $[M+Na]^{+}$

^{1}H-NMR (600 MHz, DMSO-*d6*): δ 9.65-9.45 (m, Aryl-NH-C(O)-), 8.3-8.11 (m, C(O)-NH), 8.02-7.92 (m, C(O)-NH), 7.9-7.83 (m, C(O)-NH), 7.80-7.75 (m ,1H, Triazole-H), 7.42-7.18 (m, C(O)-NH2), 7.0-6.8 (m, 8H, Aryl-H), 6.76-6.64 (m, C(O)-NH2), 4.77 (bs, $OH_{mannose}$), 4.69-4.61 (m, $OH_{mannose}$), 4.61-4.58 (m, 1H, $CH_{mannose}$), 4.56-4.39 (m, 3H, 12, $OH_{mannose}$), 4.36-4.26 (m, 4H, 14_{axial}), 3.95-3.87 (m, 1H, $CH_{mannose}$), 3.85-3.66(m, 18H, 15, 13, 8), 3.61-3.57 (m, 1H, $CH_{mannose}$), 3.56-3.53 (m, 1H, $CH_{mannose}$), 3.52-3.46 (m, 1H, $CH_{mannose}$), 3.20-3.11 (m, 4H, 3), 3.10-3.02 (m, 5H, $14_{equatorial}$, $CH_{mannose}$), 2.83 (t, ^3J = 7.2 Hz,

2H, 11), 2.78-2.69 (m, 4H, 1), 2.66-2.60 (m, 2H, 10), 2.39-2.32 (m, 16H, 16, 17), 2.3-2.22 (m, 4H, 18), 2.07 (t, ^3J = 7.4 Hz, 2H, 4), 1.92-1.82 (m, 8H, 7), 1.6-1.5 (m, 2H, 5), 1.4-1.3 (m, 2H, 6), 0.85(t, ^3J=7.3 Hz, 9H, 9) ppm.
The Signals of 2 and $CH_{mannose}$ overlap with the water peak at 3.3 ppm. Signals from unidentified impurities can be found at 1.2-1.26 ppm.

^{1}H-NMR (600 MHz, MeOH-d_4/D_2O): δ 7.85-7.69 (m, 1H, Triazole-H), 7.15-6.69 (m, 8H, Aryl-H), 4.70-4.40 (m, 1H, $CH_{mannose}$), 4.55-4.49 (m, 2H, 12), 4.44-4.36 (m, 4H, 14_{axial}), 4.08-4.01 (m, 1H, $CH_{mannose}$), 3.97-3.75 (m, 19H, 8, 15, PEG impurities), 3.74-3.70 (m, 2H, 13), 3.64-3.6 (m, 1H, $CH_{mannose}$), 3.58-3.23 (m, 2H, $CH_{mannose}$), 3.48-3.37 (m, 4H, 2), 3.36-3.3 (m, 4H, 3), 3.13-3.09 (m, 5H, $14_{equatorial}$, $CH_{mannose}$), 2.98-2.91 (m, 2H, 11), 2.80-2.73 (m, 2H, 10), 2.51-2.47 (m, 20H, 16, 17, 1), 2.44-2.40 (m, 2H, 4), 2.22-2.14 (m, 4H, 18), 1.98-1.88 (m, 8H, 7), 1.70-1.60 (m, 2H, 5), 1.48-1.38 (m, 2H, 6), 1.03-0.94 (m, 9H, 9) ppm.
Signals from unidentified impurities can be found at 1.2-1.35.

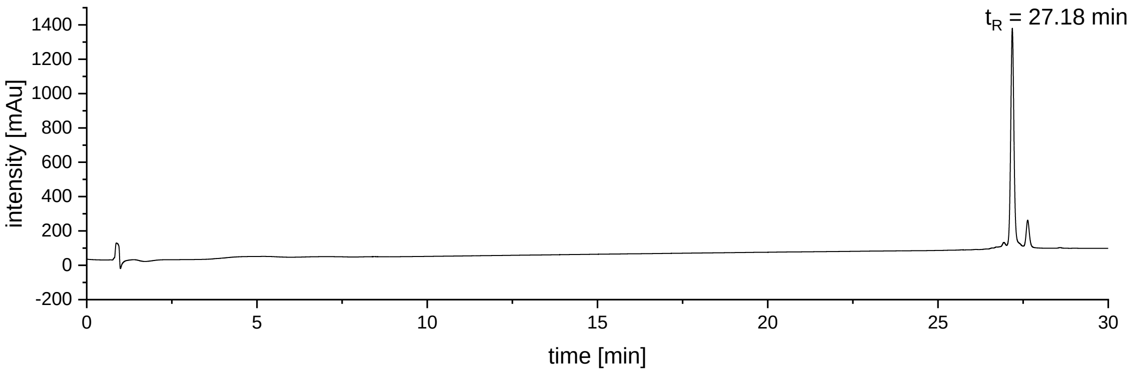

Figure S16. RP-HPLC chromatogram (100% A to 50% A in 30 min at 25° C) of compound C4.

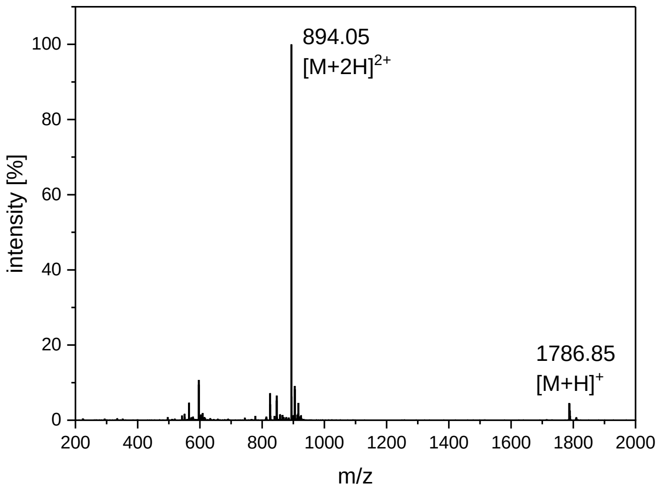

Figure S17. ESI-MS spectrum at t_R = 27.18 min (100% A to 50% A in 30 min at 25° C) of compound C4.

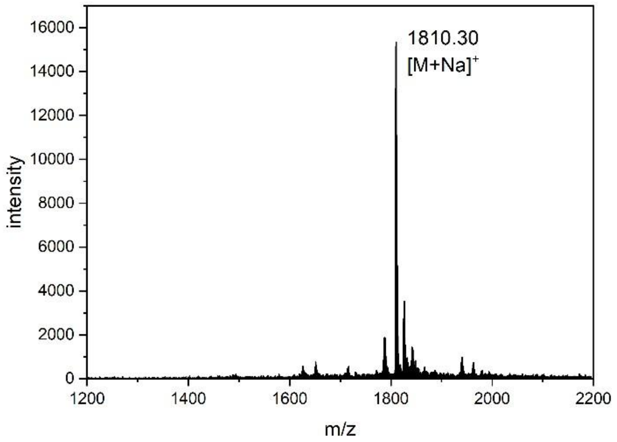

Figure S18. MALDI-TOF-MS spectrum of compound C4.

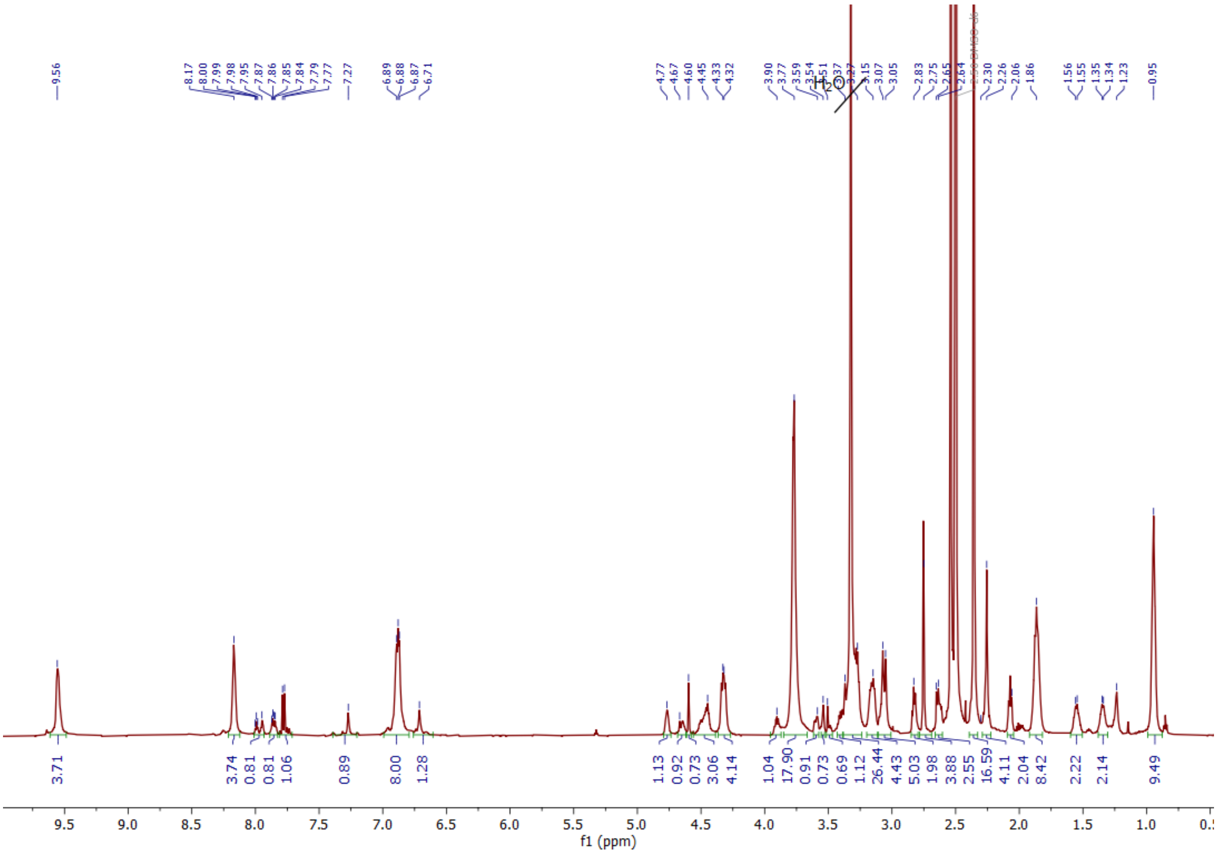

Figure S19. ^{1}H-NMR spectrum (600MHz) of compound C4 in DMSO-*d6*.

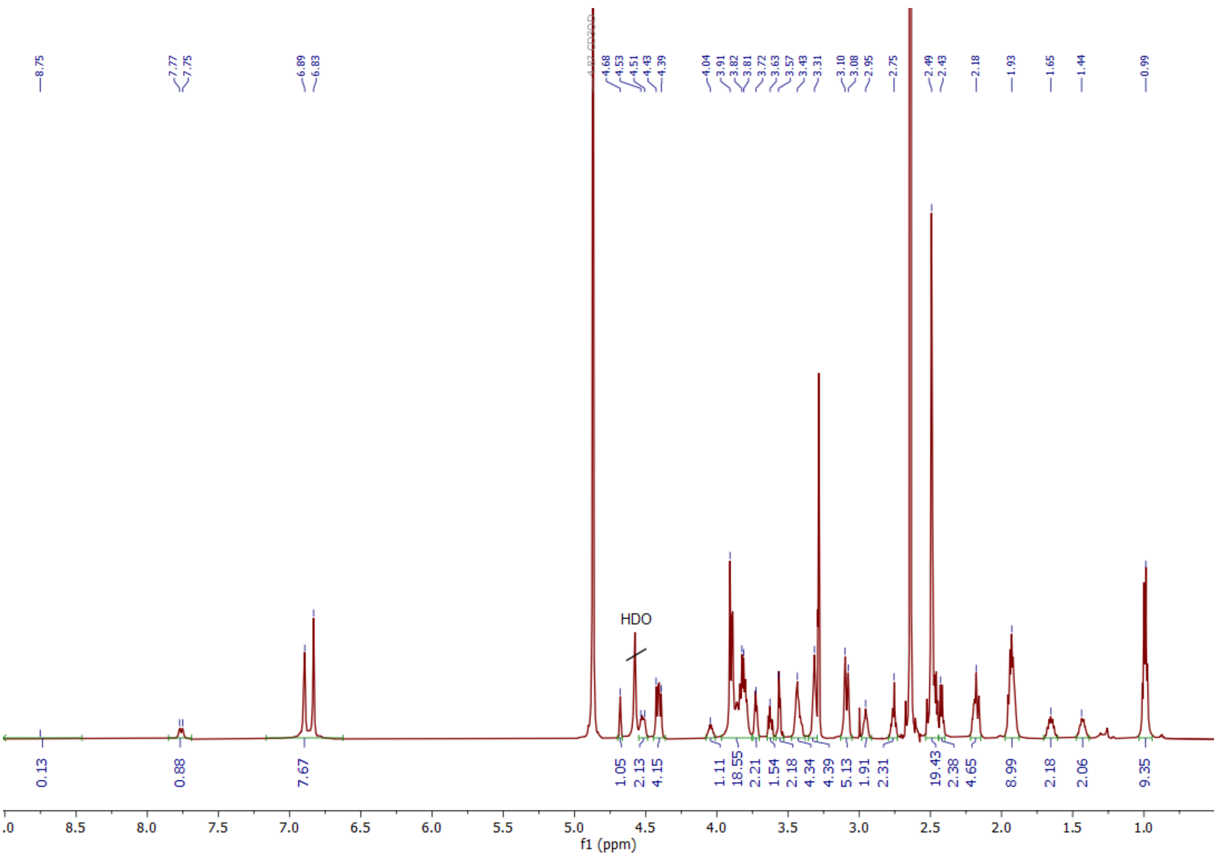

Figure S20. ^{1}H-NMR spectrum (600MHz) of compound C4 in MeOH-*d4*/D_2O.

Compound C5:

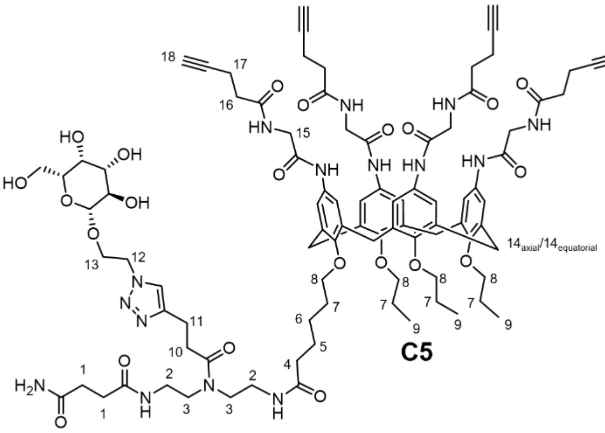

Compound **C5** was purified by preparative HPLC (62% A to 52% A in 15 min) and obtained in a yield of 42%. The relative purity of 89% was determined by integration of the UV signals of the RP-HPLC run at 214 nm.

ESI-MS: m/z calculated for $C_{92}H_{119}N_{15}O_{22}$: 1786.87 $[M+H]^{1+}$, 893.86 $[M+2H]^{2+}$, found: 1787.75 $[M+H]^+$, 894.1 $[M+2H]^{2+}$

MADLI-TOF-MS: m/z calculated for $C_{92}H_{119}N_{15}O_{22}$: 1786.87 $[M+H]^+$,1808.87 $[M+Na]^+$, found: 1810.30 $[M+Na]^+$

^{1}H-NMR (600 MHz, DMSO-d_6): δ 9.72-9.46 (m, Aryl-NH-C(O)-), 8.3-8.11 (m, C(O)-NH), 8.00-7.94 (m, C(O)-NH), 7.92-7.82 (m, 2H, Triazole-H, C(O)-NH), 7.28 (s, 1H, C(O)-NH_2), 7.00-6.78 (m, 8H, Aryl-H), 6.71 (s, 1H, C(O)-NH_2), 5.04 (s, 1H, $OH_{galactose}$), 4.86 (m, 1H, $OH_{galactose}$), 4.66 (s, 1H, $OH_{galactose}$), 4.56-4.42 (m, 3H, 12, $OH_{galactose}$), 4.37-4.26 (2d/m, 4H, 14_{axial}), 4.18-4.12 (m, 1H, $CH_{galactose}$), 4.08-3.98 (m, 1H, $CH_{galactose}$) 3.95-3.7 (m, 18H, 15, 13, 8), 3.63 (s, 1H, $CH_{galactose}$), 3.55-3.43 (m, 3H, $CH_{galactose}$) , 3.20-3.11 (m, 4H, 3), 3.06 (d, ^2J = 13.4 Hz, 4H, $14_{equatorial}$), 2.83 (t, ^3J = 7.4 Hz, 2H, 11), 2.78-2.68 (m,

4H, 1), 2.66-2.60 (m, 2H, 10), 2.4-2.31 (m, 16H, 16, 17), 2.29-2.22 (m, 4H, 18), 2.07 (t, ^3J = 7.5 Hz, 2H, 4), 1.92-1.81 (m, 8H, 7), 1.6-1,5 (p, ^3J=7.6 Hz, 2H, 5), 1.39-1.3 (m, 2H, 6), 1.00-0.9 (m, 9H, 9) ppm.

The Signals of 2 and $CH_{galactose}$ overlap with the water peak at 3.3 ppm. Signals from unidentified impurities can be found at 1.2-1.26 ppm.

^{1}H-NMR (600 MHz, MeOH-d_4/D_2O): 8.48 (bs, 1H, NH), 7.97-7.85 (m, 1H, Triazole-H), 7.11-6.7 (m, 8H, Aryl-H), 4.62-4.52 (m, 2H, 12), 4.50-4.40 (2d/m, 4H, 14_{axial}), 4.27-4.24 (dd, ^3J= 7.6, 1.8 Hz, 1H, $CH_{galactose}$), 4.24-4.18 (m, 1H, $CH_{galactose}$), 4.0-3.80 (m, 20H, 15, 13, 8), 3.78 (dd, ^2J = 11.53 Hz, ^3J = 6.99 Hz, 1H, $HOCH_{2,galactose}$), 3.72 (dd, ^2J = 11.53 Hz, ^3J = 5.08 Hz, 1H, $HOCH_{2, galactose}$), 3.56-3.33 (12H, m, 2, 3, $CH_{galactose}$, overlap with methanol peak), 3.12 (bd, ^2J = 12.56 Hz, 4H, $14_{equatorial}$), 2.99 (t, ^3J = 7.4 Hz, 2H, 11), 2.80-2.73 (m, 2H, 10), 2.58-2.39 (m, 22H, 16, 17, 1, 4), 2.25-2.19 (m, 4H, 18), 2.02-1.91 (m, 8H, 7), 1.74-1.64 (m, 2H, 5), 1.52-1.42 (m, 2H, 6), 1.08-0.98 (m, 9H, 9) ppm.

Signals from unidentified impurities can be found at 1.23-1.40 ppm.

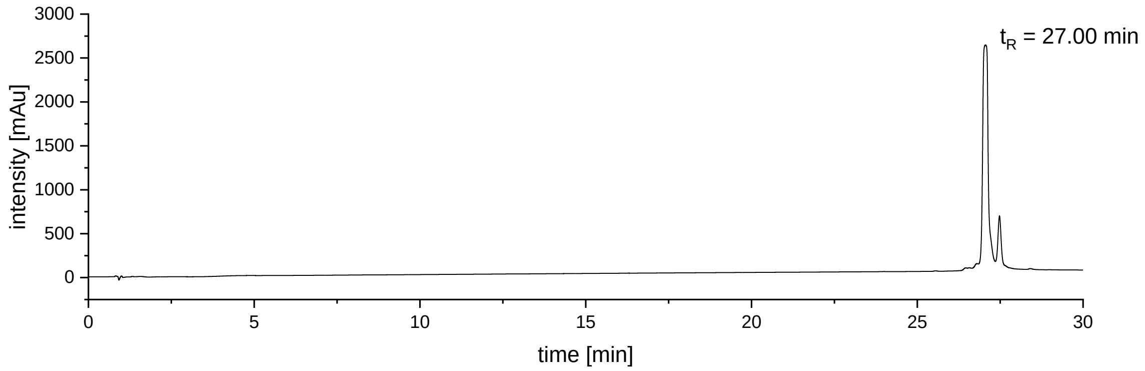

Figure S21. RP-HPLC chromatogram (100% A to 50% A in 30 min at 25° C) of compound C5.

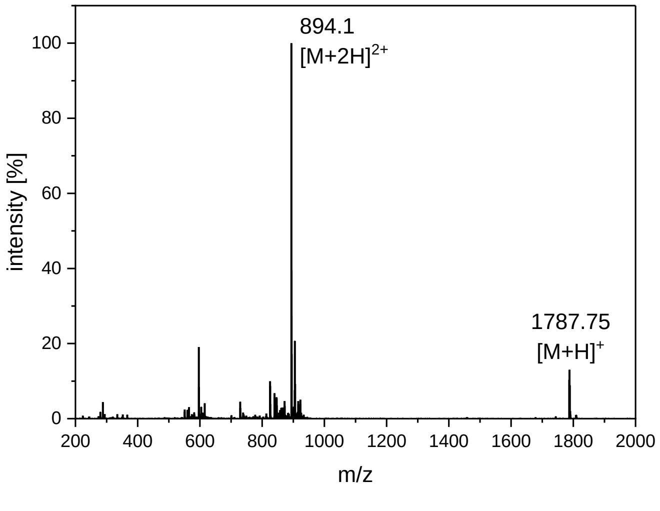

Figure S22. ESI-MS spectrum at t_R = 27.00 min (100% A to 50% A in 30 min at 25° C) of compound C5.

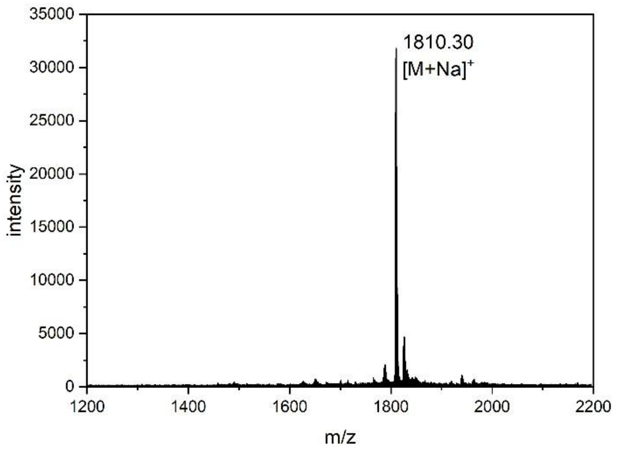

Figure S23. MALDI-TOF-MS spectrum of compound C5.

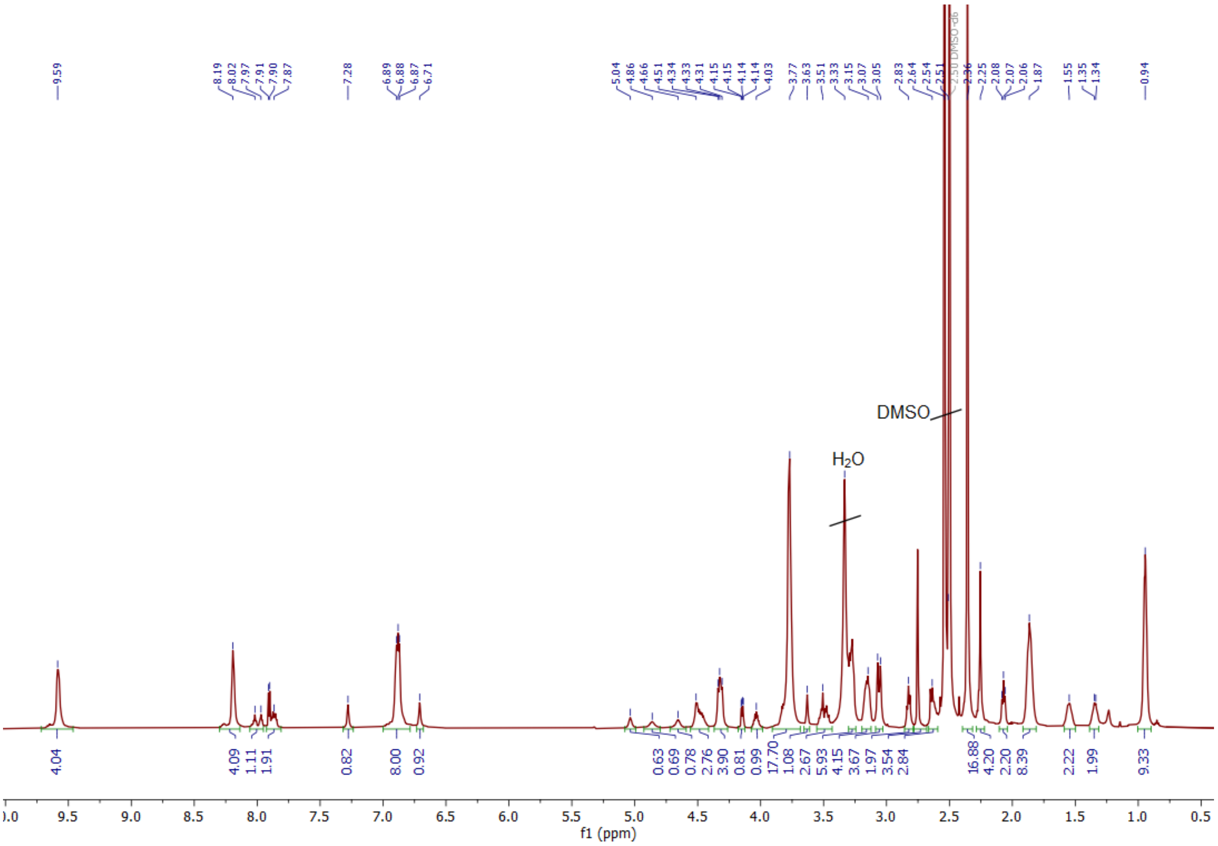

Figure S24: ^{1}H-NMR spectrum (600MHz) of compound C5 in DMSO-*d6*.

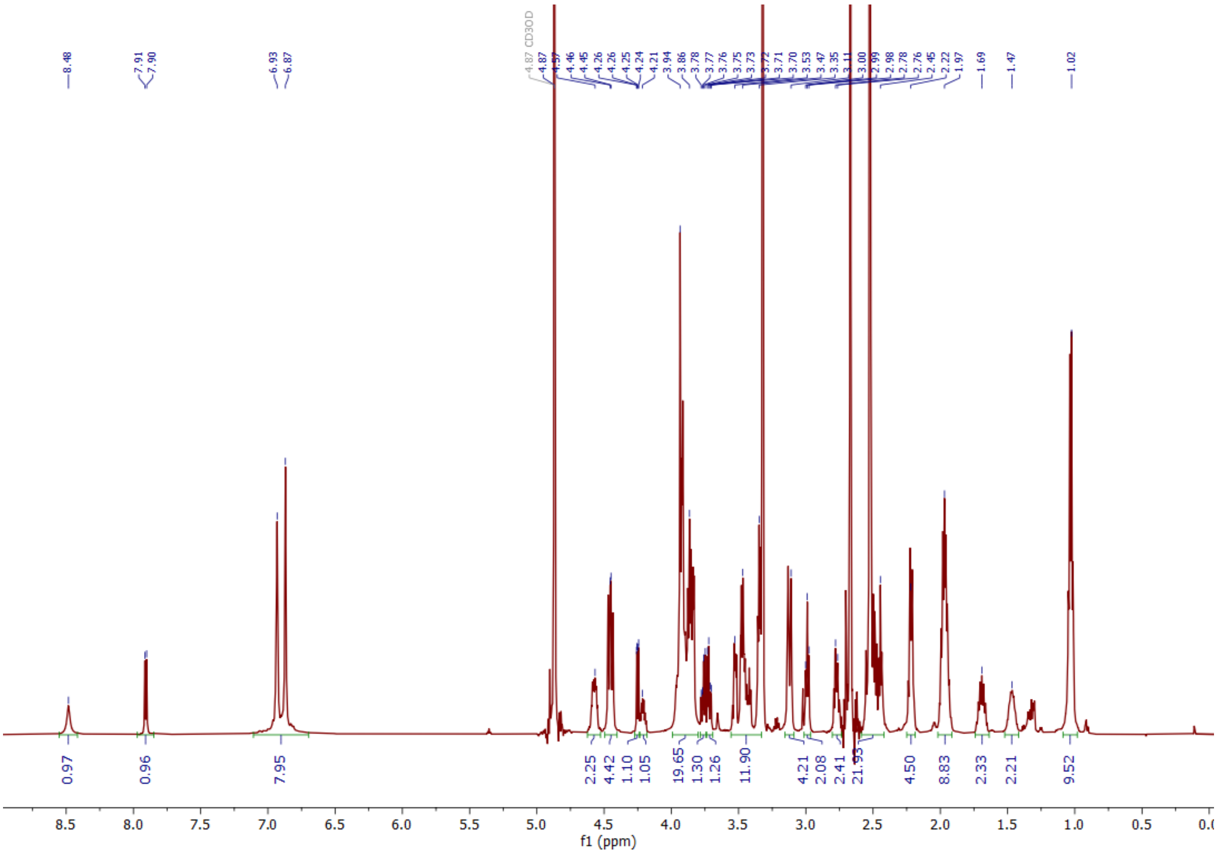

Figure S25: ^{1}H-NMR spectrum (600MHz) of compound C5 in MeOH-*d4*/D$_2$O.

Compound C6:

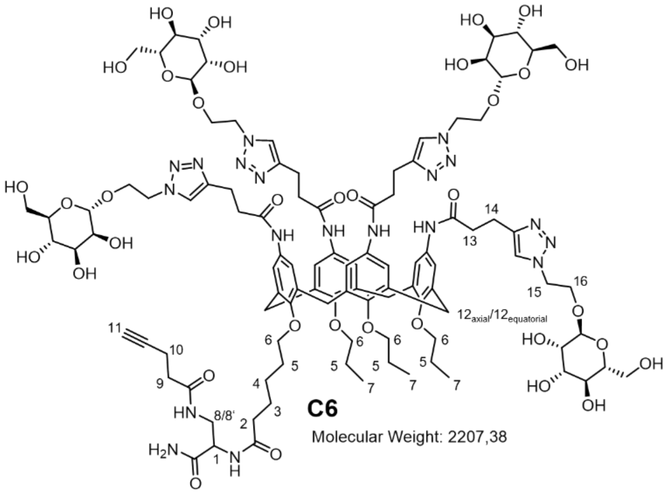

Compound **C6** was purified by preparative HPLC (70% A to 35% A in 15 min) and obtained in a yield of 65%. The relative purity of 94% was determined by integration of the UV signals of the RP-HPLC run at 214 nm.

ESI-MS: m/z calculated for $C_{92}H_{143}N_{19}O_{35}$: 1104.0 $[M+2H]^{2+}$, 736.3 $[M+3H]^{3+}$, found: 1104.55 $[M+2H]^{2+}$, 736.6 $[M+3H]^{3+}$

MALDI-TOF-MS: m/z calculated for $C_{103}H_{143}N_{19}O_{35}$: 2228.99 $[M+Na]^+$, found: 2230.18 $[M+Na]^+$

^{1}H-NMR (600 MHz, MeOH-*d$_4$*/D$_2$O): δ 8.58 (bs, -NH-), 7.73(bs, 4H, Triazole-H), 7.09-6.69 (m, 8H, Aryl-H), 4.65 (m overlap with water peak, $CH_{mannose}$), 4.43 (dd, ^3J = 8.0, 4.8 Hz, 1H, 1), 4.37 (d, ^2J = 12.75 Hz, 12_{axial}), 4.07-3.95 (m, 4H, $CH_{mannose}$), 3.87-3.38 (m, 34H, 6, 8, 8', 16, $CH_{mannose}$), 3.14-3.02 (m, 8H, $12_{equatorial}$, $CH_{mannose}$), 3.00-2.86 (m, 8H, 14), 2.7-2.51-2.7 (m,

8H, 13), 2.42-2.29 (m, 4H, 9, 10), 2.24 (t, ^3J=7.6 Hz, 2H, 2), 2.21-2.18 (m, 1H, 11), 1.98-1.81 (m, 13H, 5, overlap with acetate), 1.69-1.60 (m, 2H, 3), 1.46-1.36 (m, 2H, 4), 0.99-0.90 (t, ^3J=7.2 Hz, 9H, 7) ppm.

Signals from 15 overlap with water peak at 4.56 ppm. Acetate anions from anion exchange can be found at 1.81-1.98 ppm. Signals from unidentified impurities can be found at 1.2-1.26 ppm.

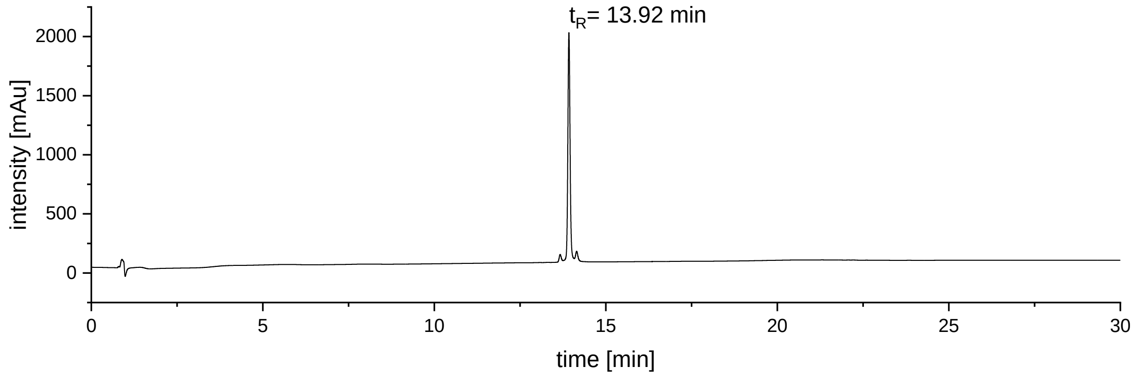

Figure S26. RP-HPLC chromatogram (100% A to 0% A in 30 min at 25° C) of compound C6.

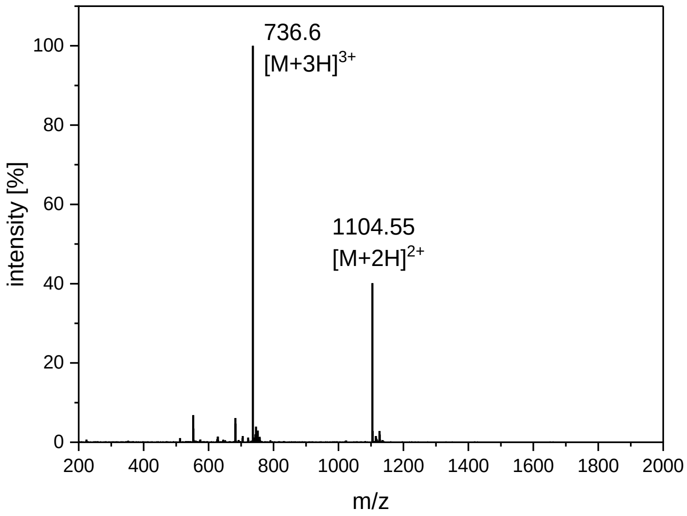

Figure S27. ESI-MS spectrum at t_R = 13.92 min (100% A to 0% A in 30 min at 25° C) of compound C6.

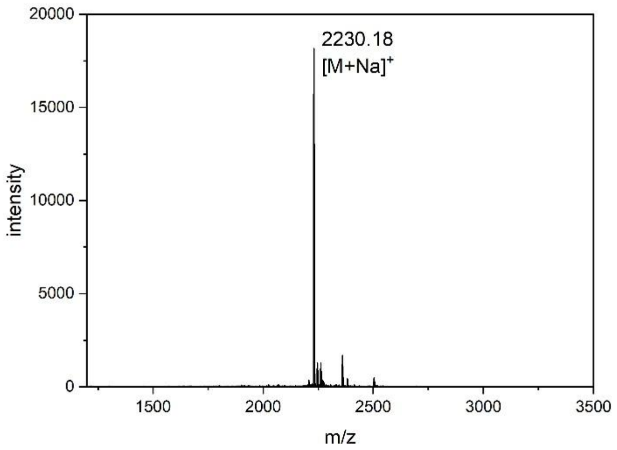

Figure S28. MALDI-TOF-MS spectrum of compound C6.

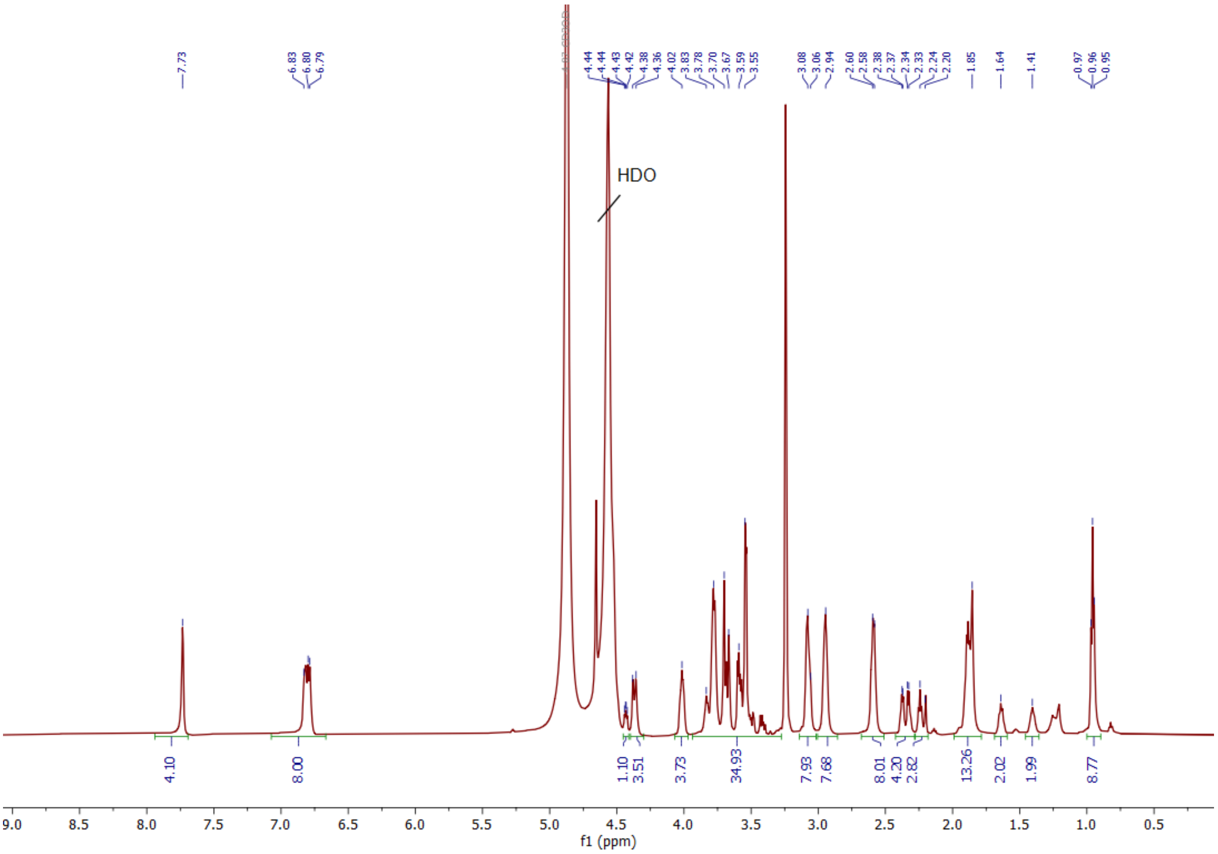

Figure S29. ^{1}H-NMR spectrum (600MHz) of compound C6 in MeOH-*d4*/D_2O.

Compound C7:

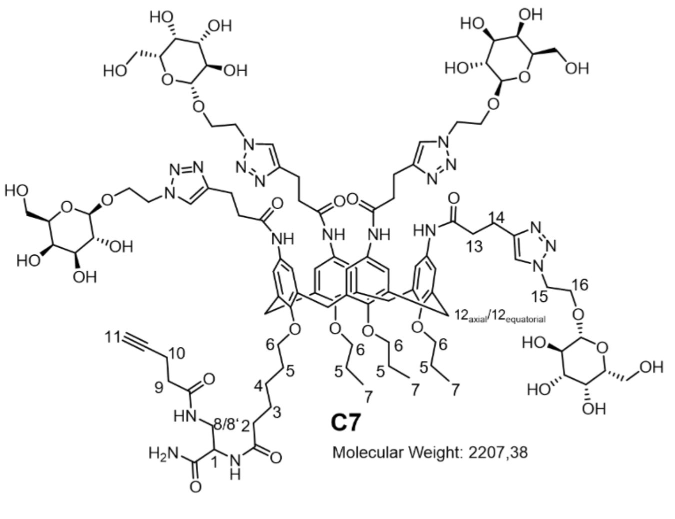

Compound **C7** was purified by preparative HPLC (70% A to 35% A in 15 min) and obtained in a yield of 62%. The relative purity of >95% was determined by integration of the UV signals of the RP-HPLC run at 214 nm.

ESI-MS: m/z calculated for $C_{92}H_{143}N_{19}O_{35}$: 1104.0 $[M+2H]^{2+}$, 736.3 $[M+3H]^{3+}$, found: 1104.3 $[M+2H]^{2+}$, 736.6 $[M+3H]^{3+}$

MALDI-TOF-MS: m/z calculated for $C_{103}H_{143}N_{19}O_{35}$: 2228.99 $[M+Na]^{+}$, found: 2230.18 $[M+Na]^{+}$

^{1}H-NMR (600 MHz, MeOH-*d4*/D_2O): δ 7.91 (s, 4H, Triazole-H), 6.94-6.77 (m, 8H, Aryl-H), 4.64-4.52 (m overlaps with water peak, 15), 4.43 (dd, ^{3}J = 7.9, 4.9 Hz, 1H, 1, overlaps with water peak), 4.37 (d, ^{2}J = 12.55, 4H, 12_{axial}), 4.31-4.11 (m, 8H, $CH_{galactose}$), 3.99-3.91 (m, 4H, $CH_{galactose}$), 3.91-3.78 (m, 12H, 6, $CH_{galactose}$), 3.75-3.66 (m, 8H, $HOCH_{2,\ galactose}$), 3.56-3.41 (m, 14H, 16H, $CH_{galactose}$), 3.15-3.07 (2d, 4H, $12_{equatorial}$), 2.97 (bs, 8H, 14), 2.75-2.52 (m, 8H, 13), 2.44-2.32 (m, 4H, 9, 10), 2.28 (t, ^{3}J=7.6 Hz, 2H, 2), 2.23 (t, ^{4}J = 2.6 Hz, , 1H, 11), 1.98-1.88 (m, 8H, 5, overlap with acetate), 1.69 (p, ^{3}J=7.7 Hz, 2H, 3), 1.49-1.41 (m, 2H, 4), 1.01 (t, ^{3}J = 7,27 Hz 9H, 7) ppm.

Signals from unidentified impurities can be found at 1.24-1.31 ppm. Acetate anions from anion exchange can be found at 2.05 ppm.

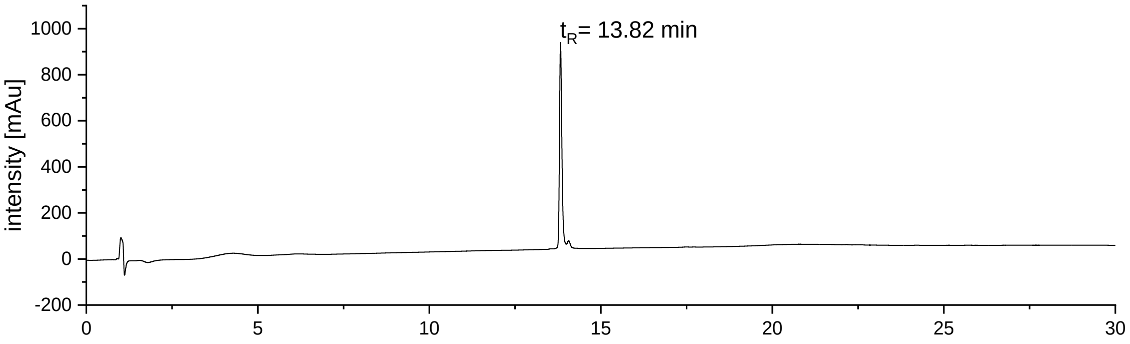

Figure S30. RP-HPLC chromatogram (100% A to 0% A in 30 min at 25° C) of compound C7.

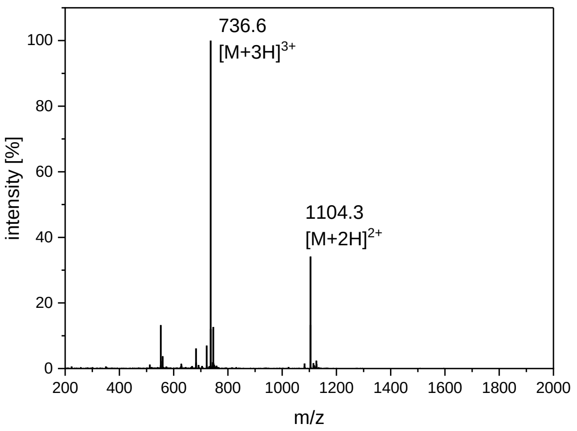

Figure S31. ESI-MS spectrum at t_R = 13.82 min (100% A to 0% A in 30 min at 25° C) of compound C7.

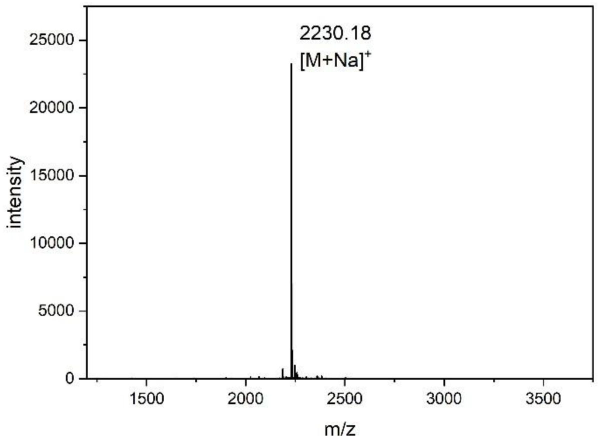

Figure S32. MALDI-TOF-MS spectrum of compound C7.

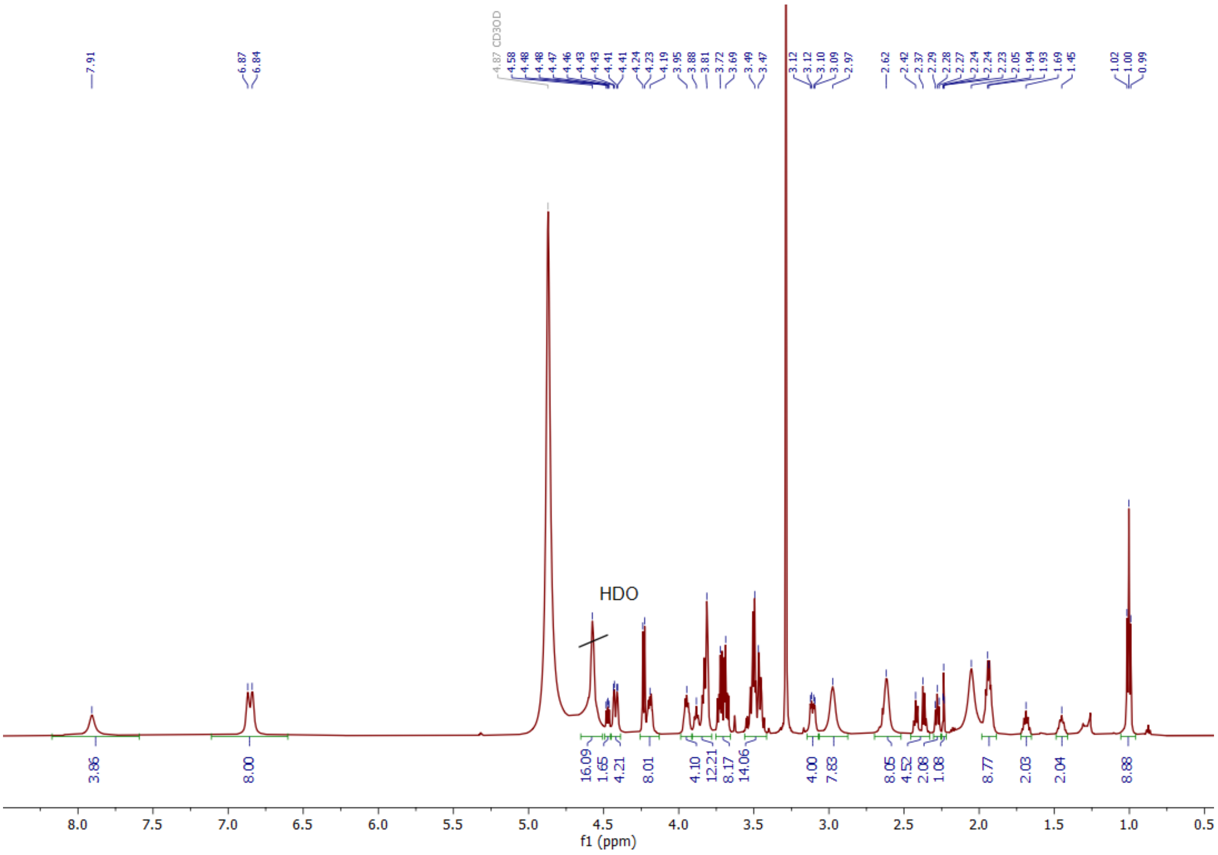

Figure S33. ^{1}H-NMR spectrum (600MHz) of compound C7 in MeOH-*d4*/D_2O.

gold nanoparticles prior to functionalization

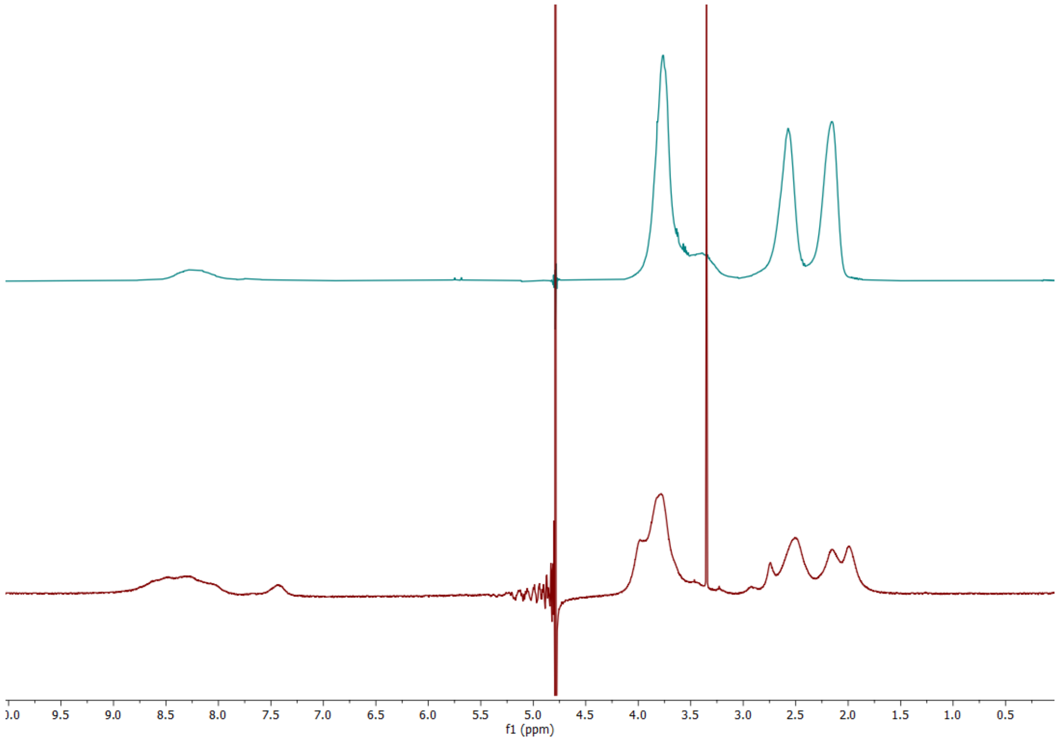

Figure S34. 1H-NMR spectra of AuGSH (blue) and AuN3 (red) in H_2O.

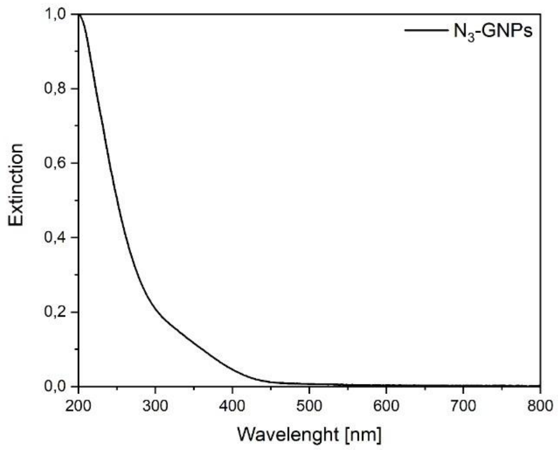

Figure S35. Normalized UV-Vis spectra of N_3-GNPs.

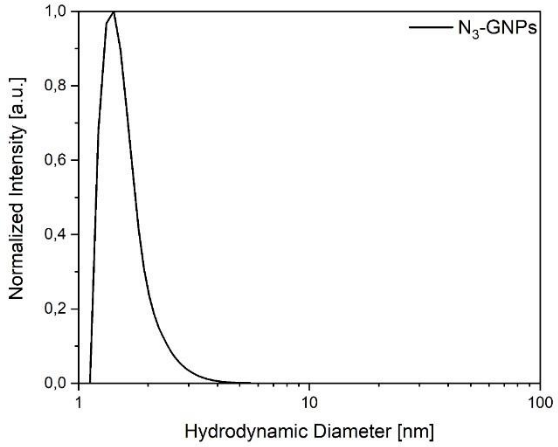

Figure S36. Disc centrifugal sedimentation (DCS) of N_3-GNPs.

glycocalixarene-gold nanoparticle conjugates

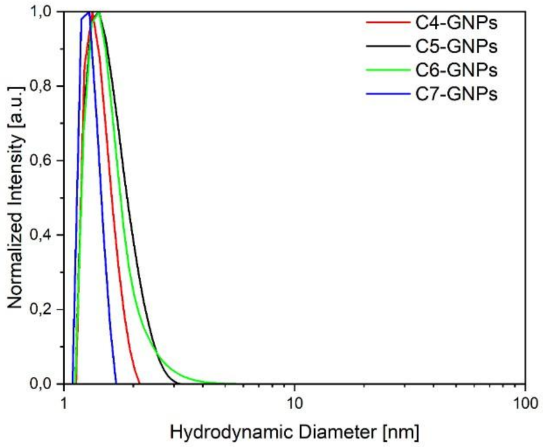

Figure S37. Disc centrifugal sedimentation (DCS) of C4-GNPs (red), C5-GNPs (black), C6-GNPs (green) and C7-GNPs (blue).

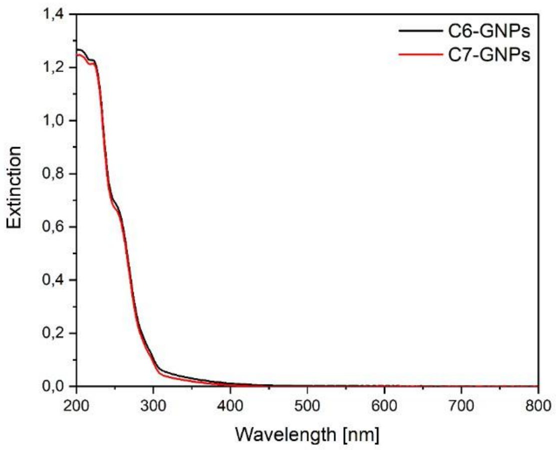

Figure S38. Normalized UV-Vis spectra of C6-GNPs (black) and C7-GNPs (red).

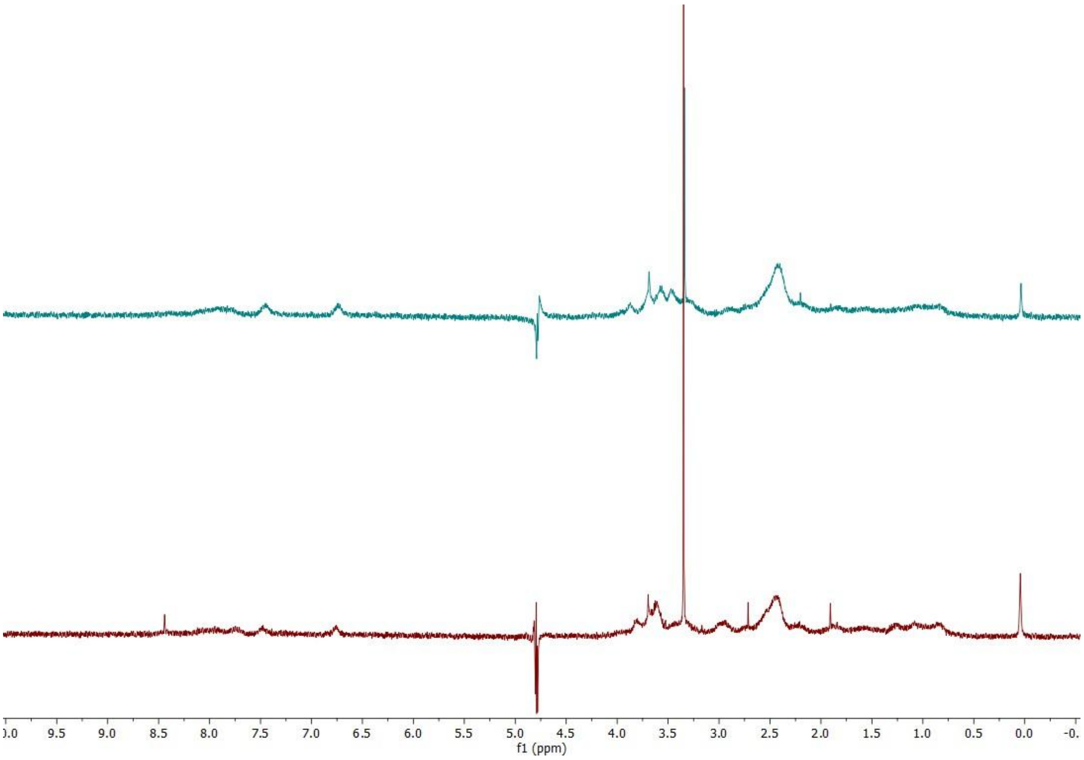

Figure S39. ^{1}H-NMR spectra of C4-GNPs (red) and C5-GNPs (blue).

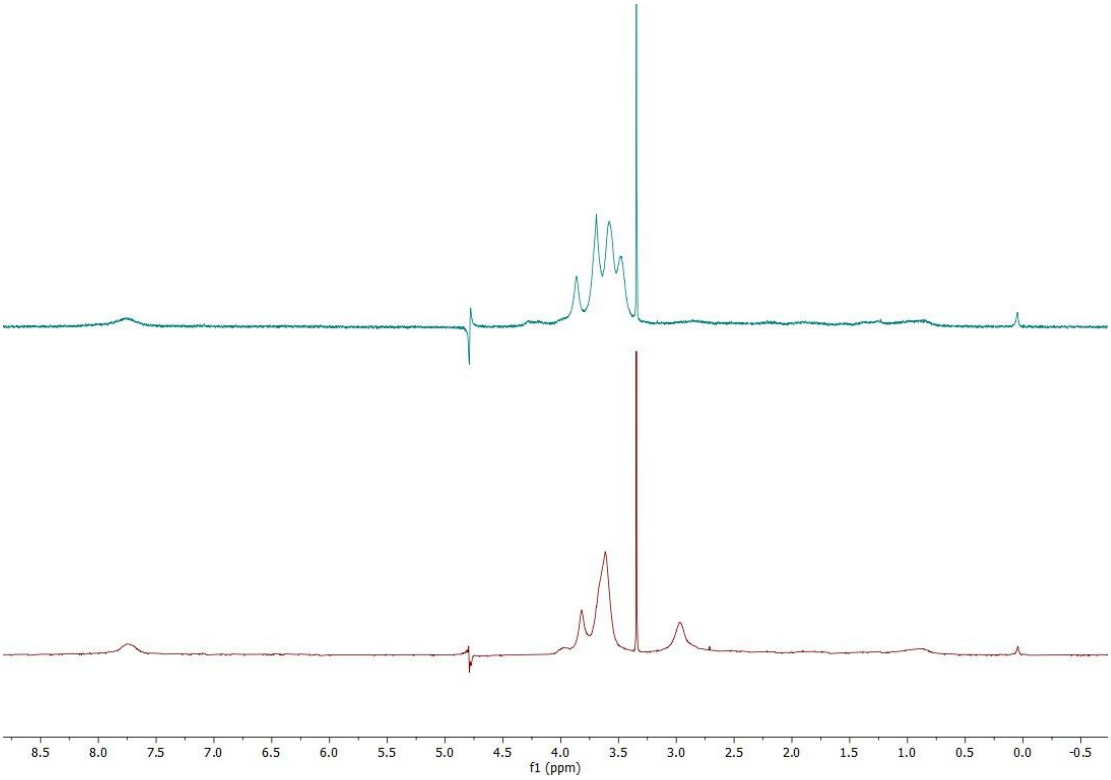

Figure S40. ^{1}H-NMR spectra of C6-GNPs (blue) and C7-GNPS (red).

Bacterial inhibition studies

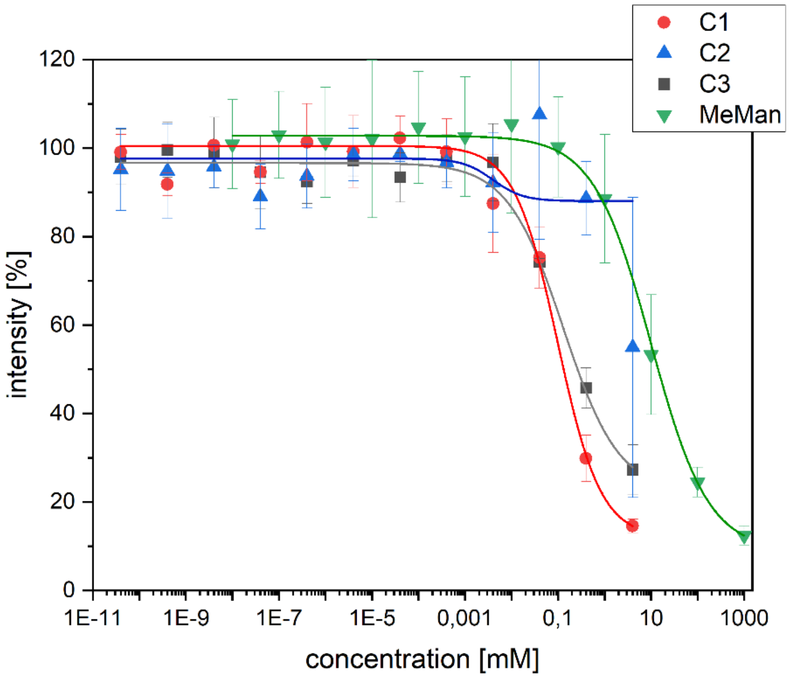

Figure S41. Bacterial adhesin inhibition assay according to protocol described above with compounds C1, C2, C3 and MeMan.

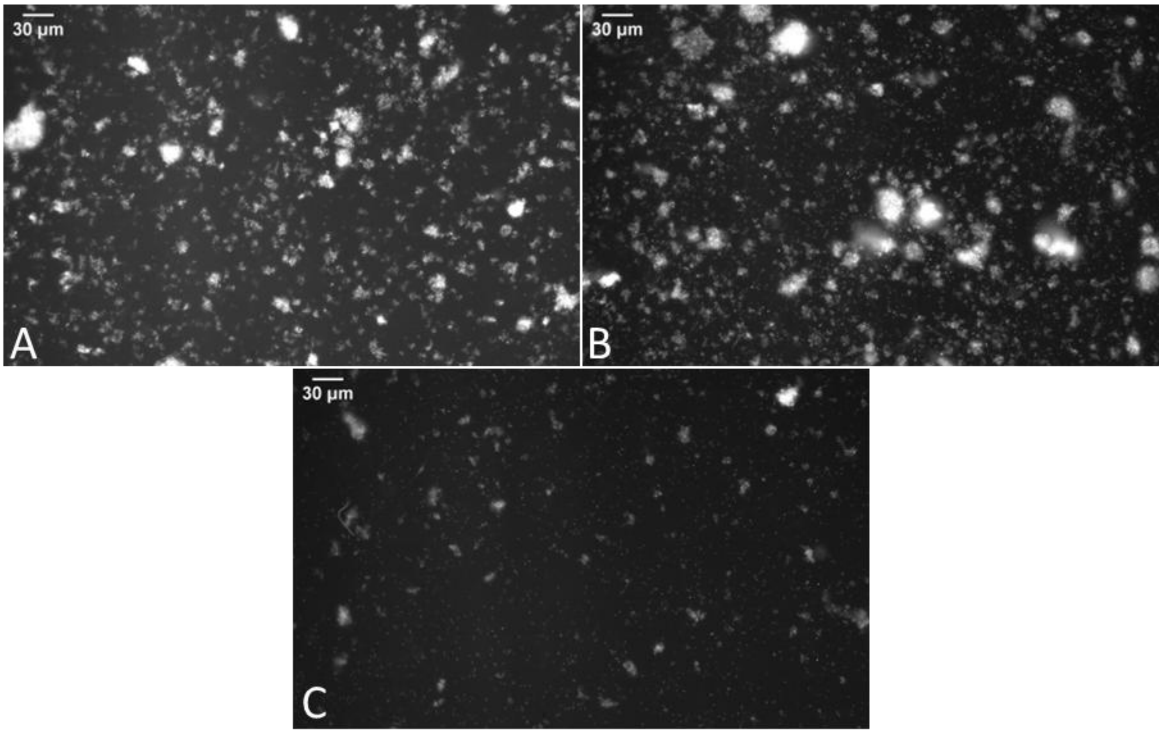

Figure S42. Fluorescence microscopy, A: C6-GNPs (c(NP) = 22.5 µM) + *E.Coli (1mg/ml)* in PBS buffer, B: *E.coli* (1mg/ml) in PBS buffer, C: C1 (c(calix[4]arene) = 22.5 µM) + *E.coli* (1mg/ml) in PBS buffer.

References:

[1] K. Klein, K. Loza, M. Heggen and M. Epple, *ChemNanoMat* **2021**, *7*, 1330-1339.
[2] a) D. Ponader, F. Wojcik, F. Beceren-Braun, J. Dernedde and L. Hartmann, *Biomacromolecules* **2012**, *13*, 1845-1852; b) M. Ebbesen, C. Gerke, P. Hartwig and L. Hartmann, *Polymer Chemistry* **2016**, *7*, 7086-7093.
[3] W. Hayes, H. M. Osborn, S. D. Osborne, R. A. Rastall and B. Romagnoli, *Tetrahedron* **2003**, *59*, 7983-7996.
[4] L. Wu and N. S. Sampson, *ACS chemical biology* **2014**, *9*, 468-475.
[5] a) S. M. Gerchakov and P. G. Hatcher, *Limnology and Oceanography* **1972**, *17*, 938-943; b) T. Masuko, A. Minami, N. Iwasaki, T. Majima, S.-I. Nishimura and Y. C. Lee, *Analytical biochemistry* **2005**, *339*, 69-72.
[6] A. K. H. Mirja Hartmann, Per Klemm, Thisbe K. Lindhorst, *Chemical Communications* **2010**, *46*, 330-332.
[7] L.-S. Wagner, O. Prymak, T. Schaller, C. Beuck, K. Loza, F. Niemeyer, N. Gumbiowski, K. Kostka, P. Bayer and M. Heggen, *The Journal of Physical Chemistry B* **2024**, *128*, 4266-4281.
[8] M. Bergeron-Brlek, T. C. Shiao, M. C. Trono and R. Roy, *Carbohydrate research* **2011**, *346*, 1479-1489.
[9] S. B. Shuker, J. Esterbrook and J. Gonzalez, *Synlett* **2001**, *2001*, 0210-0213.
